# Ecological functional near-infrared spectroscopy in mobile children: using short separation channels to correct for systemic contamination during naturalistic neuroimaging

**DOI:** 10.1117/1.NPh.11.4.045004

**Published:** 2024-10-08

**Authors:** Paola Pinti, Larisa M. Dina, Tim J. Smith

**Affiliations:** a University of London, Birkbeck, Department of Psychological Sciences, London, United Kingdom; b University College London, Department of Medical Physics and Biomedical Engineering, London, United Kingdom; c King’s College London, Department of Psychology, London, United Kingdom; d University of the Arts London, Creative Computing Institute, London, United Kingdom

**Keywords:** short separation channels, functional near-infrared spectroscopy, superficial signal regression, children, freely moving, naturalistic, systemic interferences

## Abstract

**Significance:**

The advances and miniaturization in functional near-infrared spectroscopy (fNIRS) instrumentation offer the potential to move the classical laboratory-based cognitive neuroscience investigations into more naturalistic settings. Wearable and mobile fNIRS devices also provide a novel child-friendly means to image functional brain activity in freely moving toddlers and preschoolers. Measuring brain activity in more ecologically valid settings with fNIRS presents additional challenges, such as the increased impact of physiological interferences. One of the most popular methods for minimizing such interferences is to regress out short separation channels from the long separation channels [i.e., superficial signal regression (SSR)]. Although this has been extensively investigated in adults, little is known about the impact of systemic changes on the fNIRS signals recorded in children in either classical or novel naturalistic experiments.

**Aim:**

We aim to investigate if extracerebral physiological changes occur in toddlers and preschoolers and whether SSR can help minimize these interferences.

**Approach:**

We collected fNIRS data from 3- to 7-year-olds during a conventional computerized static task and in a dynamic naturalistic task in an immersive virtual reality (VR) cave automatic virtual environment.

**Results:**

Our results show that superficial signal contamination data are present in young children as in adults. Importantly, we find that SSR helps in improving the localization of functional brain activity, both in the computerized task and, to a larger extent, in the dynamic VR task.

**Conclusions:**

Following these results, we formulate suggestions to advance the field of developmental neuroimaging with fNIRS, particularly in ecological settings.

## 1 Introduction

Functional near-infrared spectroscopy (fNIRS) is a non-invasive neuroimaging modality that measures the cortical hemodynamic and oxygenation changes that follow neuronal activity. Due to it being portable, relatively robust to head movements, and versatile for a wide range of population and tasks, fNIRS has gained increased popularity over the past 30 years, as recently acknowledged in the “Celebrating the 30 years of fNIRS” special issue in *Neurophotonics*.^1^ The tremendous progress in hardware developments has led to the availability of wireless/mobile fNIRS devices that now enable brain function imaging in real-world settings and in those situations in which other neuroimaging methods such as functional magnetic resonance imaging or electroencephalography (EEG) are not suitable, such as those requiring dynamic movements^2^^–^^4^ or face-to-face social interactions.^5^^,^^6^

fNIRS is very often used within the field of cognitive neuroscience to localize the task-evoked functional brain activity or to evaluate the brain-to-brain or within-brain functional connectivity.^7^ Results are typically assessed at the group level rather than at the individual level by testing specific hypotheses on the pooled single subjects’ data. The reliability and robustness of the inference results strongly rely on the amount of noise in the included fNIRS data. In fact, the fNIRS signals are contaminated by noise components of different origin: measurement noise (e.g., electronic noise), motion artifacts, and physiological noise (e.g., changes in blood pressure, respiration rate, and heart rate^7^^,^^8^). These can act as confounding factors in the fNIRS analysis and can either mask and/or mimic the presence of a task-evoked hemodynamic response, leading to false positives and/or false negatives in the group-level statistics.^9^ Some of these components have distinct frequency characteristics from the task-evoked hemodynamic response and hence can be easily removed or minimized using filtering methods (e.g., band-pass filter to remove very-low- and high-frequency noise or wavelet filtering to correct for motion artifacts^10^). However, other physiological confounders can overlap with the task-evoked hemodynamic activity, such as variations in arterial blood pressure known as Mayer waves (∼0.1  Hz^11^) or respiration (∼0.2  Hz), and more advanced methods are needed to minimize their impact.

Among these, one major source of interference on the cortical fNIRS signals comes from extra-cerebral contamination: the near-infrared light emitted from the light source travels twice through various layers of the head (skin, skull, dura, and cerebrospinal fluid) before reaching the brain and being back-scattered to the detector.^9^ Previous work has shown that ∼96% of the injected light is absorbed in the skin and skull, whereas only 3% is absorbed in the brain.^12^ Therefore, the measured fNIRS signal is a mixture of the hemodynamic and oxygenation changes happening in both the superficial and cortical vasculature. In addition, blood flow changes in both the brain and the scalp can be modulated by those physiological processes that act on the vascular tone. These processes encompass changes in blood pressure, respiration, partial pressure of carbon dioxide (${\mathrm{PaCO}}_{2}$), and autonomic activity, among others. These changes can either be spontaneous (i.e., heartbeat, respiration, variations in arterial blood pressure or Mayer waves, and very low frequencies), modulated, or induced by the task itself. For example, posture changes can alter blood pressure,^13^ speaking and jaw movements can lead to changes in ${\mathrm{PaCO}}_{2}$ and non-neural blood flow changes related to the use of the temporalis muscle,^14^ and stressful tasks can promote vasoconstriction by triggering a sympathetic response.^15^ Even passive and apparently stress-free tasks such as passive color light exposure can induce physiological changes.^16^ It is thus reasonable to expect even a larger impact of systemic interferences when fNIRS neuroimaging experiments are extended from the stationary laboratory setting to those with a higher degree of ecological validity in which participants may be able to walk, engage in dynamic movements, or speak freely.^17^

Different strategies have been proposed so far to deal with physiological interferences, such as multimodal monitoring or appropriate task design (e.g., avoid 10-s-long task and rest blocks that overlap with Mayer waves),^7^ including experimental conditions with the same level of physical activity so that some of the effects can be subtracted when contrasting the conditions (see Burgess et al.^3^ for an example). A popular method to account for superficial contamination is to use short separation channels alongside long separation channels. In contrast with long separation channels in which a pair of source and detector is placed at a distance >2  cm (typically 2 cm for infants and 3 cm for adults) and is assumed to be sampling from the brain, short separation channels are created by placing a source and a detector at a <1-cm distance and assuming that the back-scattered photons have traveled through the superficial layers of the head only and are not brain-sensitive.^18^ The optical signal from the short separation channel can be regressed out from the long separation channel to obtain a more brain-specific fNIRS signal with reduced scalp contamination. This method is often referred to as superficial signal regression (SSR^19^^,^^20^). SSR has been widely used in the fNIRS field and has been demonstrated to improve the recovery of the hemodynamic response,^21^ even in instances when strong Mayer wave components are present.^11^ It was also proven to be effective in increasing the performance of resting-state functional connectivity analyses.^22^

Although the benefit of SSR has been well established in adult populations and nowadays can be considered common practice,^7^ less is known on its impact on the group-level statistics of developmental fNIRS data. Furthermore, it is not routinely used in children’s fNIRS data analysis. It is reasonable to hypothesize that the scalp hemodynamic changes might be different in younger populations compared with adults because of a different head anatomical structure or maturation stage of the vasculature system;^23^ therefore, it is not clear yet whether there are significant superficial changes in children and whether regressing out the short separation channels can reduce physiological interference and increase the reliability of the task-evoked group level inferences. To date, only the study by Ferradal et al.^24^ performed SSR in high-density fNIRS data recorded on newborns. However, others have reported task-evoked patterns in channels with a source–detector separation of ∼10  mm in 4- to 7-month-old infants that suggest that a proportion of this signal is of superficial origin.^23^^,^^25^ Emberson et al.^23^ were the first to investigate whether SSR has an impact on the group-level statistics in a developmental sample; however, this was only investigated in infants’ fNIRS data. They showed that both superficial and deeper hemodynamic responses can be observed in the occipital cortex of 6-month-old babies while undergoing a visual and auditory stimulation task. However, the removal of superficial signals did not lead to significant changes in the group-level results. As pointed out by the authors, these results might be task-specific, region-specific, and population-specific, and a different outcome may be expected in other experiments or populations; thus, further investigations are needed.

There remains a notable gap in knowledge regarding the benefits of SSR across development, such as in toddlers and preschoolers. Previous studies have shown that cerebral blood flow is lower in the postnatal brain than in adults, then increases until 7 years of age, and then decreases to a similar level to adults in teenage years, which may reflect brain maturation and synapse formation.^26^ Therefore, children older than 3 years old may exhibit a different pattern of blood flow changes which may significantly differ from those of younger or older population. More importantly, task-evoked hemodynamic changes in these age groups may be modulated by experimental contexts (e.g., standing or sitting) and physical movements, as previously shown in adults.^13^^,^^17^ With the recent advances in wireless and more mobile NIRS instrumentation, cognitive neuroscience investigations can now move to more naturalistic settings,^27^ which are more dynamic and may thus enhance the impact of physiological interferences on the fNIRS-derived brain signals. Increasing the ecological validity of fNIRS procedures is of critical importance for studying toddlers and preschoolers whose neuro-cognitive development is traditionally under-researched due to their difficulty sitting still and complying with task demands.

In this work, we aim to fill this gap and investigate if (1) there are significant extracerebral physiological and hemodynamic changes in toddlers and preschoolers, (2) extracerebral interference is stronger when fNIRS data are recorded in standing and freely moving children, (3) SSR can mitigate the impact of systemic confounding factors and improve the robustness of fNIRS data, and (4) SSR has a significant effect on the group-level inference results on the task-evoked brain activity. To this goal, we collected fNIRS data on a group of 3- to 7-year-old children undergoing an inhibitory control task, a core executive function.^28^ Participants performed a standard computer version of the task sitting at a desk and a naturalistic virtual reality (VR) version adapted after the computer task. The VR task was carried out in an immersive VR cave automatic virtual environment (CAVE) where kids could stand and were able to move about. The task was designed to account for possible systemic interferences. Finally, we provide some recommendations on how to adapt some of the best practices^7^ to help enable fNIRS neuroimaging on young children.

Our hypotheses are as follows: (1) there are task-evoked changes in scalp blood flow in toddlers and preschoolers; (2) these are larger in the VR version of the task compared with the computer-based one because of posture (standing versus sitting) and physical activity; (3) SSR changes the outcome of the group level statistics, especially in the VR task; and (4) SSR has a larger effect on oxygenated hemoglobin than deoxygenated hemoglobin, as previously found in adults.^9^^,^^29^

## 2 Material and Methods

### 2.1 Participants

Thirty-nine 3- to 7-year-old children ($M_{\text{age}}=4.45$, SD=1.08, 35.9% female) were recruited. All participants were born full-term, healthy, with normal or corrected to normal vision and hearing, and with no diagnosis of a neurodevelopmental condition. The protocol for this study was preregistered on the Open Science Framework.^30^ All parents or caregivers provided written informed consent. Ethical approval was granted by the Ethics Committee of the Department of Psychological Sciences at Birkbeck, University of London (No. 2021072).

For the VR task, seven participants were excluded for poor fNIRS data and two for task performance; for the computer task, two participants were excluded due to poor fNIRS data and performance, six for poor fNIRS data only, and one for task performance only. The final sample thus included 30 participants for the VR task ($M_{\text{age}}=4.5$, SD=1.14, 21 males) and 30 participants for the computer task ($M_{\text{age}}=4.53$, SD=1.14, 21 males).

### 2.2 Experimental Protocol

Participants performed a response inhibition task, a type of inhibitory control referring to our ability to suppress a certain action or a prepotent response. The go/no-go task is a widely used measure of response inhibition, which requires participants to press a button in response to some stimuli and to refrain from button presses when certain other stimuli appear.^28^ Here, we used child-friendly versions of a go/no-go task.^31^ In particular, a block-designed go/no-go task was performed in two versions: a computerized version and a VR version.

#### 2.2.1 Computer-based (CB) go/no-go task

**Fig. 1:**
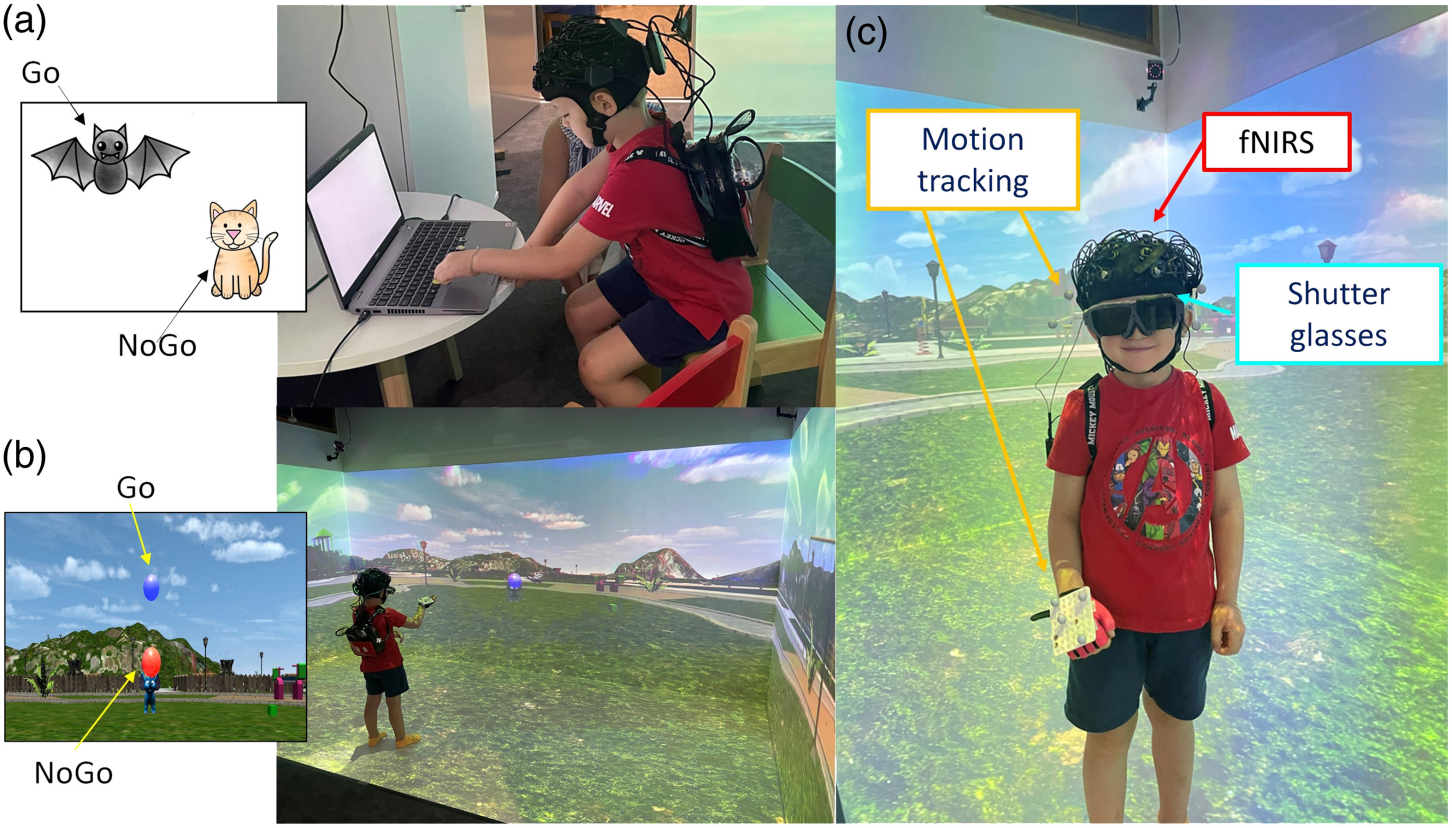
Experimental protocol. Children performed a computerized version of the go/no-go task sitting in front of a computer (a) and a VR version while freely moving in a VR CAVE (b). In the CAVE, children wore the fNIRS cap in conjunction with motion tracking markers and custom-made shutter glasses.

This version [Fig. 1(a)] was previously used in Schröer et al.^31^ and restructured as a block-design to fit with fNIRS requirements. Children were sitting in front of a computer screen and were told a story that a town was haunted by vampires and that they had to be monster hunters and catch all of the bats that would soon become vampires. They were presented with pictures of bats (go trials) and cats (no-go trials). They were asked to press the space bar on the computer keyboard as fast as possible to catch the bats (go) but refrain from doing so when they would see a cat (no-go). The task started with two practice trials before moving on to the main experiment.

#### 2.2.2 VR go/no-go task

The VR version of the task was performed in the Peltz VR CAVE at the ToddlerLab, Birkbeck, University of London [Fig. 1(b)]. The CAVE is an immersive VR space made of a four-sided custom-designed projection system (Mechdyne Corporation, Marshalltown, Iowa, United States). The projection space includes a front wall (4.3×2  m), two side walls (2.4×2  m), and the floor (4.3×2  m). Two blended single-chip laser projectors overlapping by 65% (resolution of 2716×1528  pixels; total resolution=3297×1528  pixels) display images onto the front and floor walls; a single laser projector (resolution 2716×1528  pixels) is used to present images on the side walls. Children wore an in-house-built three-dimensional (3D) printed model of a child-sized pair of liquid crystal display (LCD) shutter classes for active stereo viewing at 30 Hz that increases the immersive experience of participants in the virtual scene. Four six-degree-of-freedom optical motion tracking cameras (Vero 1.3 X, Vicon, Hauppauge, New York, United States) at the corners of the CAVE tracked the orientation and movements of the children’s head and right hand. Head tracking was achieved by reflective markers mounted onto the shutter glasses [Fig. 1(c)] and allowed for the virtual scene to be reoriented according to the participant’s position. Reflective markers were also attached to a child-sized glove to track the right hand movement, which was used as a means of interaction with the virtual objects. The right hand was tracked for all participants regardless of handiness as the task did not require fine motor skills. The same glove was presented in the virtual environment, and the position was updated in real time based on the participant’s hand position and movements.

In this version, participants found themselves in a virtual playground. An elephant-shaped bubble machine was located at the center of the playground and generated virtual bubbles of two different colors: blue (go trials) and red (no-go trials). Children were asked to pop the blue bubbles (go) but not the red bubbles (no-go trials) by means of the motion-tracked glove. The task started with practice trials to help the child acclimated to the virtual space and ensure the child was comfortable and confident in how to use their right hand to pop the bubbles.

Both the VR and CB go/no-go tasks were block-designed, with six go-only blocks and six go/no-go (mixed; 50% go trials and 50% no-go trials) blocks. A total of 120 trials were split into 90 go-only trials and 30 no-go trials across the blocks. Each block had between 9 and 11 trials each; each trial was presented on screen or the CAVE for 2 s with an intertrial interval of a maximum of 1 s. Task blocks were spaced by rest periods with a randomized duration between 8 and 12 s; in the computer-based task, children were asked to look at a fixation cross at the center of the screen. To replicate this in the CAVE, they were asked to fixate a star appearing on the elephant’s trunk. Each task took between 6 and 8 min to complete. Go-only and mixed blocks were alternated. The order of the CB and VR tasks was counterbalanced across participants.

### 2.3 fNIRS Data Acquisition

**Fig. 2:**
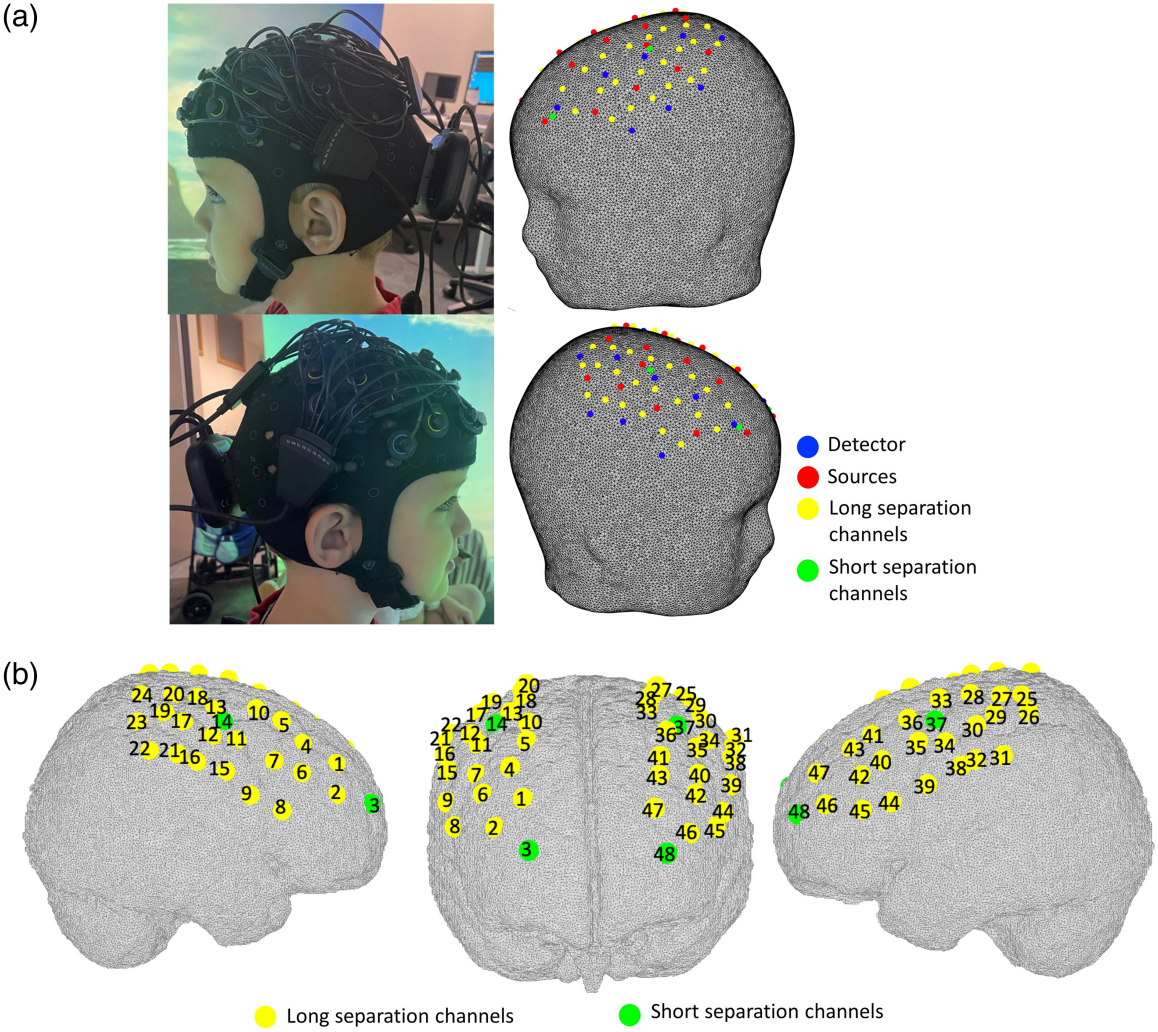
fNIRS probe configuration. Optodes’ arrangement (a) and corresponding channel numbers (b). Short and long separation channels are marked in green and yellow, respectively.

Two wearable and wireless continuous wave fNIRS devices (Brite MKII, Artinis Medical Systems BV, Elst, Netherlands) were combined onto the same cap to measure the concentration changes of oxygenated hemoglobin (${\mathrm{HbO}}_{2}$) and deoxygenated hemoglobin (HbR) while children carried out both the VR and the CB go/no-go tasks. Each instrument is equipped with 10 light sources, emitting light at 760 and 840 nm, and eight detectors, sampling intensity data at 25 Hz. Optodes were arranged in the configuration shown in Fig. 2(a), providing 44 long separation channels (LSCs) and 4 short separation channels (SSCs) [Fig. 2(b)].

Sources and detectors covered the dorsolateral prefrontal cortex (dlPFC) and the motor cortex bilaterally (Fig. 2). The source–detector separation for the LSCs was set at 2.5 cm and 1 cm for the SSCs. The source–detector separation of the SSC was constrained by the geometry of the optodes of the fNIRS hardware, which did not allow the sources and detectors to be placed at less than 1 cm away. Based on Brigadoi and Cooper^18^, the optimum source–detector distance for SSCs resulted in being 0.84 cm for adults and 0.215 cm for infants, suggesting that 1 cm might include some signal contribution from the brain in toddlers and preschoolers. To investigate the age-related differences in channels’ brain sensitivity for SSCs at 1 cm, we performed a preliminary analysis on the sensitivity distribution of our fNIRS array in younger (3-year-olds) and older (5-year-olds) children’s head models. This analysis demonstrated that SSCs at 1 cm are minimally sensitive to the brain in 5-year-olds but still include some brain contribution in 3-year-olds (Fig. S1 in the Supplementary Material), and a source–detector separation of 0.8 cm might be more appropriate (Fig. S2 in the Supplementary Material). Methodological procedures and further details about the computation of the sensitivity distributions are included in Sec. 1 of the Supplementary Material.

To achieve a reliable placement across participants, the cap was aligned to the Fp1 and Fp2 landmarks of the 10 to 20 electrode placement system. A short 10-s-long frontal video of the participant was recorded to perform the co-registration of the fNIRS array onto a common template (see Sec. 2.4).

### 2.4 fNIRS Data Preprocessing and Analysis

For each participant, the locations of the optodes and channels were co-registered onto a common 5-year-old MRI template from the Neurodevelopmental MRI Database of the University of South Carolina^32^^,^^33^ using the procedure described in Ref. 34. Briefly, we 3D printed the MRI template to create our head model and to specify the ideal placement of the cap; the head model coordinates of the sources and detectors as well as the anatomical landmarks (Nasion, Inion, Cz, right and left preauricular points, Fp1, Fp2, Fpz, F7, F8, O1, and O2) were recorded using a 3D magnetic digitizer (FasTrak, Polhemus, Colchester, Vermont, United States). These were used as an input to STORM-Net (https://github.com/yoterel/STORM-Net) for the off-line stage of the algorithm. The frontal videos of the participants were then used for the online step to estimate the position of the child’s optodes and landmarks based on the displacement with respect to the ideal head model.^35^ The MRI template was segmented into five tissues [scalp, skull, cerebrospinal fluid, grey matter (GM), and white matter] using the FMRIB Software Library (FSL^36^), and a volumetric multilayer mask was then created using routines from the DOT-HUB toolbox (https://github.com/DOT-HUB). A tetrahedral volumetric mesh and a GM surface mesh were generated; the optodes’ coordinates for each participant were co-registered onto the scalp mesh first via affine transformation and then projected onto the GM surface mesh. The same procedure was applied to the head model coordinates. The LONI Probabilistic Brain Atlas (LPBA40^37^) atlas was used on the head model data to identify the anatomical locations of all channels; the anatomical labels of the channels and their percentage of overlap are reported in Table 1.

**Table 1:** Anatomical locations of the fNIRS channels. The anatomical areas (LPBA40 atlas) and the corresponding atlas-based probabilities for each channel are included. Only probabilities greater than 20% are listed. Number of participants contributing to each channel for the VR ($N_{\mathrm{VR}}$) and CB ($N_{\mathrm{CB}}$) tasks because of cap placement and data quality are also listed.

Channel	LPBA40 label	Probability	$N_{\mathrm{VR}}$	$N_{\mathrm{CB}}$
Ch 1	R middle frontal gyrus	0.84	28	24
Ch 2	R middle frontal gyrus	0.88	29	28
Ch 3	R middle frontal gyrus	0.99	25	24
Ch 4	R middle frontal gyrus	0.86	20	20
Ch 5	R superior frontal gyrus	0.65	19	13
	R middle frontal gyrus	0.35		
Ch 6	R middle frontal gyrus	0.96	30	27
Ch 7	R middle frontal gyrus	0.93	26	25
Ch 8	R inferior frontal gyrus	0.76	27	30
Ch 9	R precentral gyrus	0.75	25	26
Ch 10	R superior frontal gyrus	0.75	17	15
	R middle frontal gyrus	0.24		
Ch 11	R precentral gyrus	0.70	27	23
Ch 12	R postcentral gyrus	0.57	25	22
	R precentral gyrus	0.32		
Ch 13	R superior frontal gyrus	0.48	18	16
	R precentral gyrus	0.45		
Ch 14	R precentral gyrus	0.78	13	14
Ch 15	R postcentral gyrus	0.48	28	27
	R precentral gyrus	0.34		
Ch 16	R supramarginal gyrus	0.64	26	26
	R postcentral gyrus	0.31		
Ch 17	R postcentral gyrus	0.61	22	22
Ch 18	R precentral gyrus	0.61	14	15
	R postcentral gyrus	0.30		
Ch 19	R superior parietal gyrus	0.45	21	20
	R postcentral gyrus	0.38		
Ch 20	R postcentral gyrus	0.48	14	16
	R superior parietal gyrus	0.37		
Ch 21	R supramarginal gyrus	0.89	24	23
Ch 22	R angular gyrus	0.55	26	25
	R supramarginal gyrus	0.40		
Ch 23	R superior parietal gyrus	0.56	23	25
	R angular gyrus	0.40		
Ch 24	R superior parietal gyrus	0.96	19	21
Ch 25	L superior parietal gyrus	0.91	15	17
Ch 26	L angular gyrus	0.42	24	27
	L supramarginal gyrus	0.35		
Ch 27	L superior parietal gyrus	0.49	11	16
	L postcentral gyrus	0.47		
Ch 28	L precentral gyrus	0.58	14	15
	L postcentral gyrus	0.35		
Ch 29	L postcentral gyrus	0.57	16	20
	L superior parietal gyrus	0.22		
Ch 30	L postcentral gyrus	0.71	13	16
Ch 31	L supramarginal gyrus	0.93	21	22
Ch 32	L supramarginal gyrus	0.83	23	22
Ch 33	L precentral gyrus	0.50	13	14
	L superior frontal gyrus	0.37		
Ch 34	L precentral gyrus	0.53	17	17
	L postcentral gyrus	0.43		
Ch 35	L precentral gyrus	0.57	22	21
	L middle frontal gyrus	0.37		
Ch 36	L middle frontal gyrus	0.76	13	11
Ch 37	L precentral gyrus	0.70	15	15
	L middle frontal gyrus	0.21		
Ch 38	L postcentral gyrus	0.62	28	26
	L supramarginal gyrus	0.21		
Ch 39	L precentral gyrus	0.55	25	26
	L postcentral gyrus	0.37		
Ch 40	L middle frontal gyrus	0.90	28	27
Ch 41	L middle frontal gyrus	0.85	19	15
Ch 42	L middle frontal gyrus	0.95	28	26
Ch 43	L middle frontal gyrus	0.94	23	18
Ch 44	L inferior frontal gyrus	0.48	20	26
	L precentral gyrus	0.38		
Ch 45	L inferior frontal gyrus	0.70	22	30
	L middle frontal gyrus	0.29		
Ch 46	L middle frontal gyrus	0.83	28	28
Ch 47	L middle frontal gyrus	0.97	25	22
Ch 48	L middle frontal gyrus	0.95	22	20

**Fig. 3:**
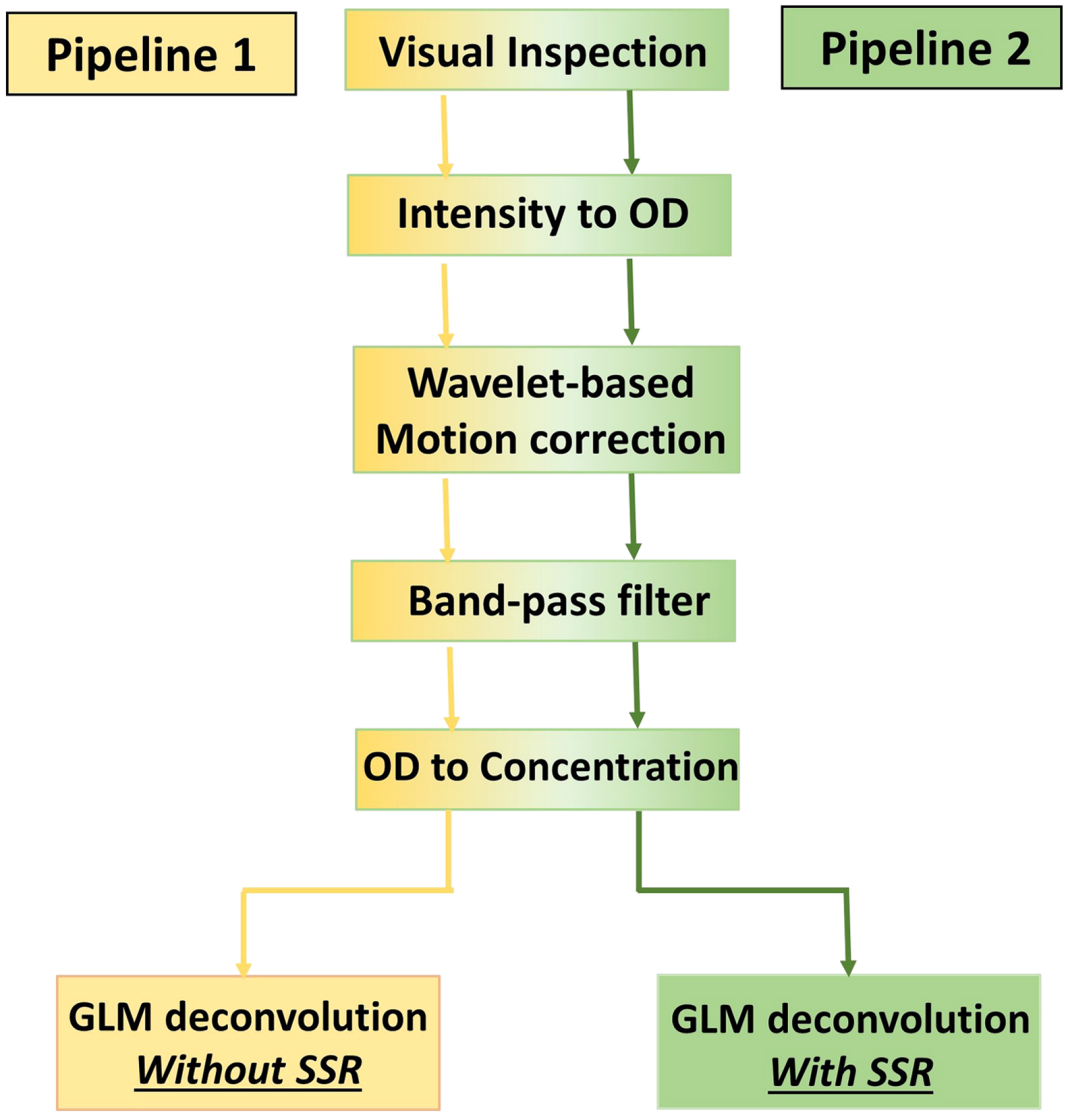
Analysis pipelines.

Raw intensity fNIRS data were first visually inspected to identify noisy channels to further exclude from the following analyses; channels with no clear heart rate peak or detector saturation or that were severely impacted by motion artifacts were excluded. Table 1 includes the number of participants that contributed good-quality data for each channel. Intensity signals were then preprocessed using Homer2.^38^ More precisely, two separate pipelines were applied to both the VR and CB tasks (Fig. 3) to compare the impact of SSR.

In both pipelines, raw fNIRS data were first converted into changes in optical density (*hmrIntensity2OD*) and corrected for motion artifacts using the wavelet-based algorithm (iqr = 0.8, *hmrMotionCorrectWavelet*^39^). Optical density signals were band-pass-filtered (Fc = [0.01 0.1] Hz; *hmrBandpassFilt*) and converted into changes in ${\mathrm{HbO}}_{2}$ and HHb through the modified Beer–Lambert law (DPF = 5.5, 4.7; *hmrOD2Conc*^40^).

Participants were included in the analysis if they had at least 50% of the fNIRS channels of good quality and at least three blocks with a performance >50% for both the go-only and mixed blocks. A general linear model-based deconvolution approach was then used to estimate the hemodynamic responses to the go and mixed blocks, for each participant, channel, and chromophore. The hemodynamic responses were extracted using a set of Gaussian basis functions with standard deviation and temporal spacing of 1.5 s in the time window [−2  32]  s around each onset of the included blocks; for pipeline 2, the design matrix included the short separation channel with the highest correlation to each long separation channel;^41^ no SSC regression was performed in pipeline 1 (trange = [− 2 32], glmSolveMethod = 1, idxBasis = 1, paramsBasis = [1.5 1.5], rhoSD_ssThresh = 0 for pipeline 1 and 1.5 cm for pipeline 2, flagSSmethod = 1, driftOrder = 0, and flagMotionCorrect = 0; *hmrDeconvHRF_DriftSS*). In this case, a local SSR regression (i.e., regressing out the highest correlated SSC for each LSC) was chosen over a global SSR regression (i.e., regressing out the average of all SSCs out of all LSCs) to account for the heterogeneity of scalp interference; in addition, as our array did not cover the whole head and included a small number of SSCs (up to four), the average of all SSCs might not reflect a truly global component (i.e., signal changes that affect the whole brain or large regions). This was confirmed by our supplementary analyses using pipeline 2 with global SSR regression (trange = [−2 32], glmSolveMethod = 1, idxBasis = 1, paramsBasis = [1.5 1.5], rhoSD_ssThresh = 1.5 cm, flagSSmethod = 2, driftOrder = 0, and flagMotionCorrect = 0; *hmrDeconvHRF_DriftSS*); in fact, results showed that the global SSR regression leads to very minor to no differences compared with the local SSR at the group level (see Tables S1–S4 and Figs. S3 and S4 in the Supplementary Material).

For both the VR and CB tasks, the area under the curve (AUC) in the estimated responses to the go and mixed blocks were computed within a time window from 15 to 25 s post-task onset; this was done for each pipeline and for both ${\mathrm{HbO}}_{2}$ and HHb of each participant and was used for the group-level statistics. Channel-wise one-sample t-tests were run on the group AUCs for each pipeline to test whether there is a statistically significant (p<0.05) larger increase in ${\mathrm{HbO}}_{2}$ and a larger decrease in HbR in the mixed blocks versus the go blocks. One-sample t-tests were also used to test whether there were significant hemodynamic changes in the SSCs in response to either the mixed or the go blocks. Results were corrected for multiple comparisons using the false discovery rate (FDR^42^) method.

## 3 Results

### 3.1 Optimization of the Preprocessing Pipeline

**Fig. 4:**
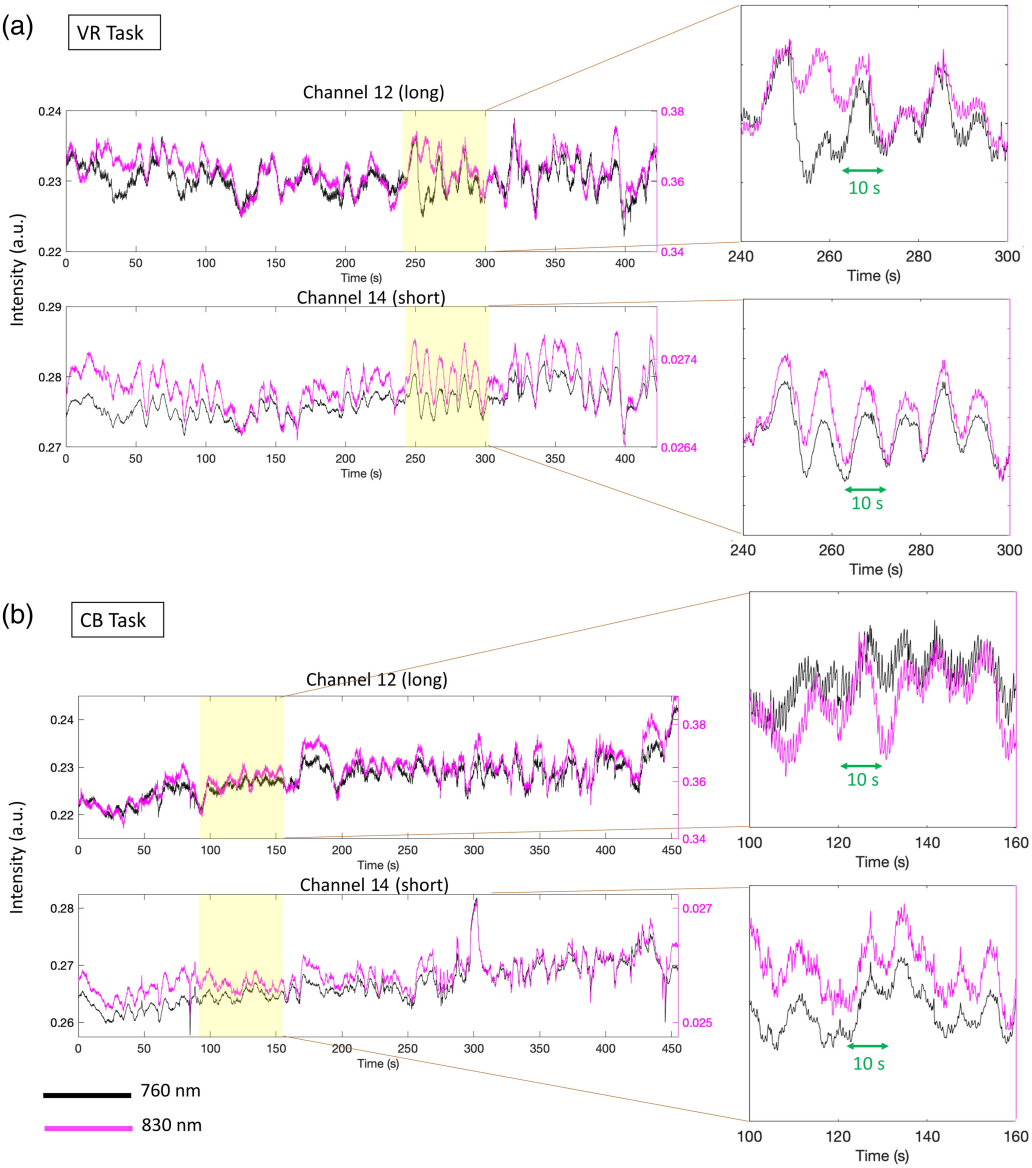
Examples of raw fNIRS data. Panels (a) and (b) show the raw intensity signals at the two wavelenghts for long and short separation channels of one participant for the VR task (a) and the CB task (b). Periodic 10-s components corresponding to Mayer waves can be observed in all channels.

Initial visual inspection of the raw intensity data revealed that some children exhibited signal changes with a periodicity of 10 s corresponding to Mayer waves, similar to what has been reported in adults.^11^ These were clearly visible from the raw intensity time series, both in the long separation channels and in the short separation channels. In Fig. 4, we show an example of raw signals at the two wavelengths for a long (channel 12) and a short (channel 14) separation channel for one child. In addition to the heartbeat, the Mayer wave component can be clearly distinguished for the VR task [Fig. 4(a)] and, to a lesser extent, for the CB task [Fig. 4(b)], suggesting that Mayer waves may be present when fNIRS data are recorded from children both when they are standing and when they are sitting.

**Fig. 5:**
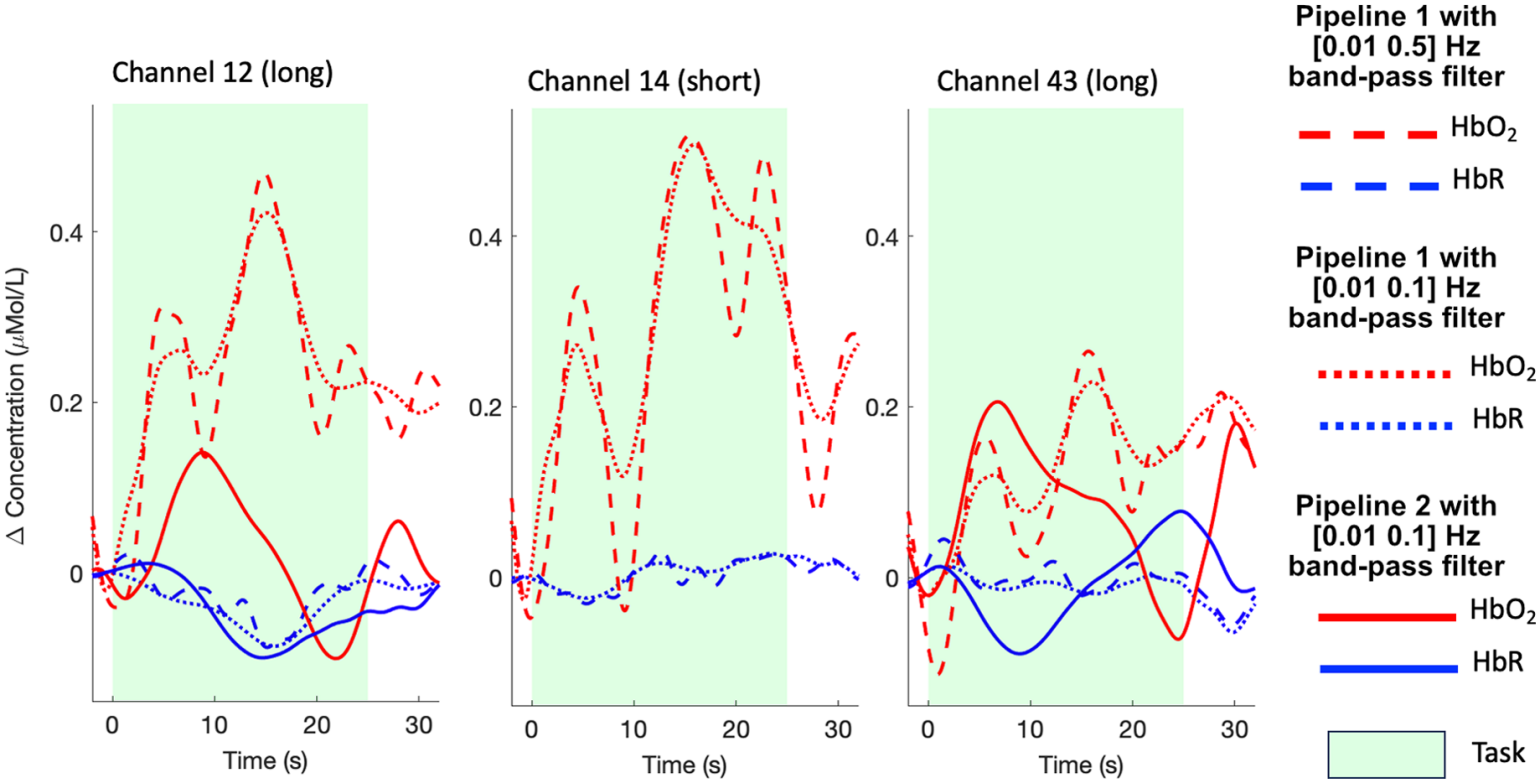
Impact of different pipelines on the reduction of physiological components. The estimated task-evoked hemodynamic responses with different pipelines are presented for the go-only condition for two long channels (channel 12 adjacent to channel 14 and channel 43 on the opposite hemisphere) and a short separation channel (channel 14) from one participant. The use of a band-pass filter only in pipeline 1, either with a wider ([0.01 0.5] Hz; dashed lines) or narrower ([0.01 0.1] Hz; dotted lines) pass band are not as effective as the combination of SSR with a narrower band-pass filter ([0.01 0.1] Hz; solid lines) in pipeline 2 in reducing the Mayer wave modulations in the signals.

When using common low pass cutoff frequencies of 0.5 Hz^7^ in conjunction with a high pass filter at 0.01 Hz in pipeline 1, the 10-s modulations related to the Mayer waves are still visible in the block-averaged responses, especially for ${\mathrm{HbO}}_{2}$. Figure 5 (dashed lines) shows the resulting hemodynamic responses averaged across the VR go blocks for channel 12 and channel 14 presented in Fig. 4(a). Channel 43 is included as well to show the effect of the three pipelines on a long separation channel not adjacent to channel 14. It is important to notice that similar hemodynamic changes occur both in channels 12 and 14, with amplitude changes of ∼0.5  μMol/L for ${\mathrm{HbO}}_{2}$ and that similar modulations are present in the opposite hemisphere as well (channel 43).

However, when reducing the pass band of the filter to [0.01 0.1] Hz, such modulations are attenuated (Fig. 5, dotted lines). Therefore, even though Mayer waves cannot be simply removed by low pass filters,^11^ a low pass cutoff frequency closer to the Mayer waves fundamental frequency (0.1 Hz) can help in attenuating the effect of such components. It is worth mentioning that these parameters are appropriate for our task design with a task frequency of ∼0.03  Hz, with the [0.01 0.1]-Hz range including three harmonics of the task frequency. In the case of faster task designs, careful considerations should be made in narrowing the pass band of the filter.^8^ When performing short channel regression following the [0.01 0.1]-Hz filter in pipeline 2, the Mayer waves modulations are further reduced (Fig. 5, solid line), particularly in ${\mathrm{HbO}}_{2}$, suggesting that, for our type of experimental context and population, the combination of these preprocessing steps seems to be optimal in reducing the impact of physiological noise. At the group level, for the VR task, this translates to either inflation or deflation of t-statistics for the contrast of interest (mixed > go-only). Group-level statistical results are reported in Tables S1–S2 and Fig. S3 in the Supplementary Material. Results suggest that leaving all or part of the Mayer wave modulations in the signals can either mask or mimic the presence of a hemodynamic response.^9^ This seems to be more pronounced in ${\mathrm{HbO}}_{2}$ for which more differences in group-level statistics between pipeline 2 and pipeline 1 (with either filter range) are observed in 11 channels compared with HbR, for which three channels are affected. In addition, pipeline 2 maximizes the overlap among the significantly active channels for ${\mathrm{HbO}}_{2}$ and HbR (i.e., concurrent and anticorrelated changes in the two chromophores are expected in the case of a functional brain activity), further suggesting that SSR combined with a narrower band-pass filter can increase the robustness of the data. In terms of the CB group-level statistics (Tables S3–S4 and Fig. S4 in the Supplementary Material), differences between pipeline 2 and pipeline 1 (with either filter range) are found in one channel for ${\mathrm{HbO}}_{2}$ and two channels for HbR, indicating that the presence of Mayer waves in the signals affects group-level statistics in stationary tasks to a lesser extent than mobile and dynamic tasks.

### 3.2 Extracerebral Changes and Impact of SSR

**Fig. 6:**
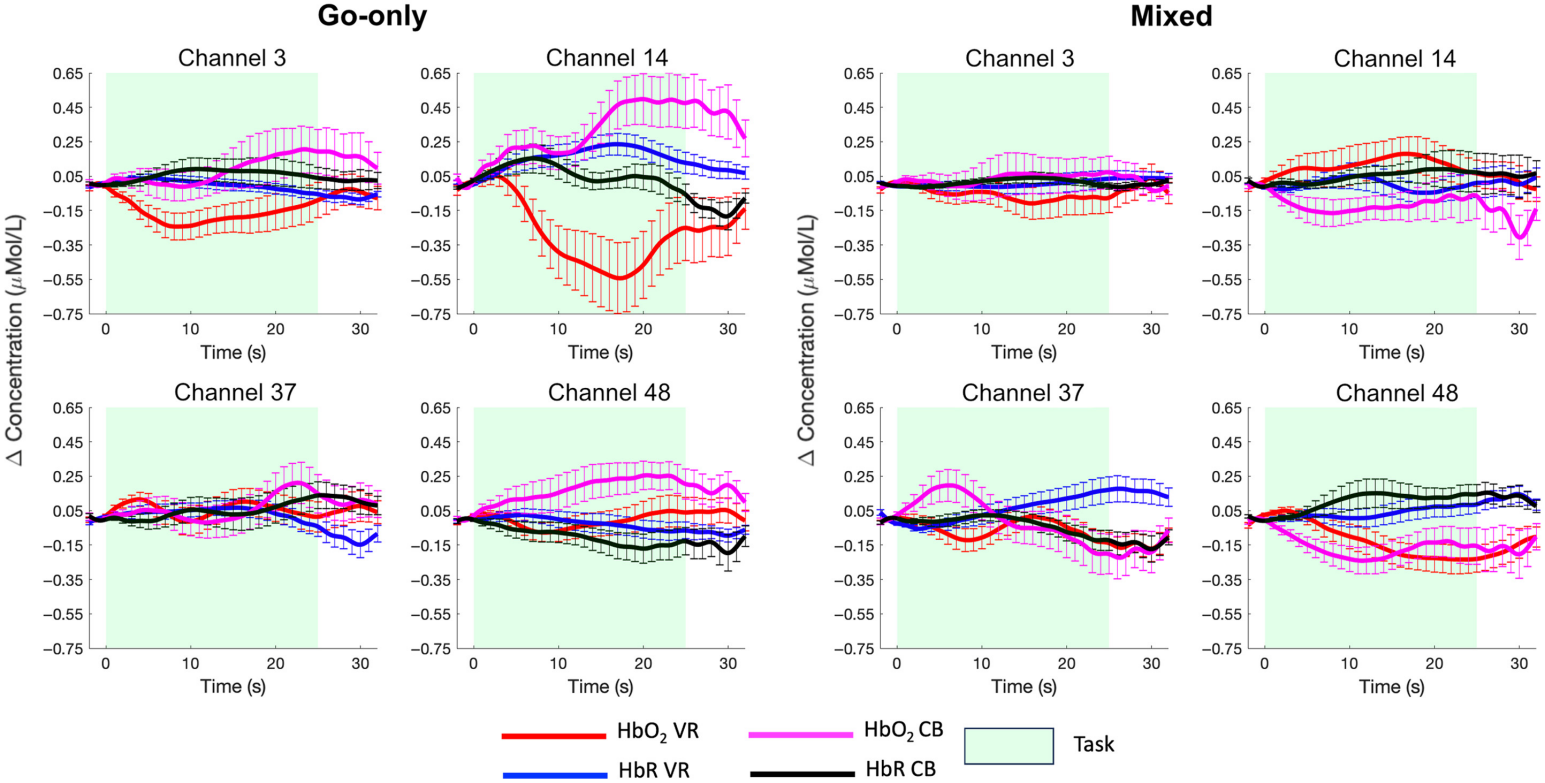
Group averaged (mean ± std.err.) ${\mathrm{HbO}}_{2}$ and HbR responses in the short separation channels. Task-evoked hemodynamic responses are shown for both the go-only and mixed conditions and for the VR (${\mathrm{HbO}}_{2}$ red and HbR blue) and CB (${\mathrm{HbO}}_{2}$ magenta and HbR black) tasks.

At the group level, significant changes were found in the short separation channels (Fig. 6). For the VR task, we found a significant decrease in ${\mathrm{HbO}}_{2}$ in channel 48 ($t_{\mathrm{ch}48}=-2.54$; p=0.02 uncorrected) for the mixed condition. For the CB task, a significant increase in ${\mathrm{HbO}}_{2}$ in channel 14 ($t_{\mathrm{ch}14}=2.27$; p=0.04 uncorrected) and channel 48 ($t_{\mathrm{ch}48}=2.36$; p=0.03 uncorrected) was found for the go-only condition. When comparing mixed > go-only, the increase in ${\mathrm{HbO}}_{2}$ is larger in the go-only condition than in the mixed blocks. All statistical results are included in Table S5 in the Supplementary Material.

**Fig. 7:**
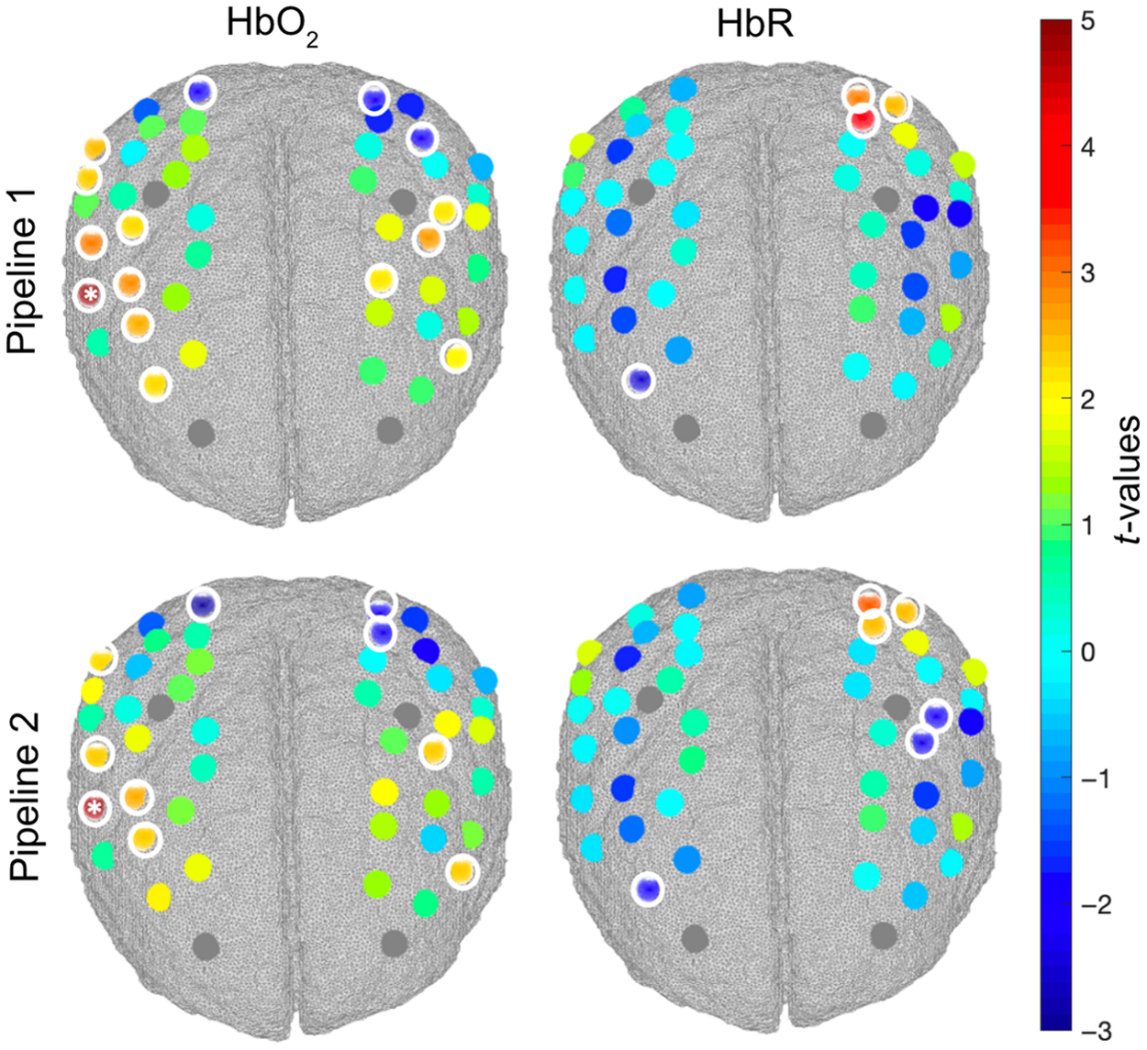
Group level t-value maps for the contrast mixed > go-only for the VR task. Statistically significant channels at p<0.05 are circled in white. Channels surviving FDR correction are marked with asterisks. A positive t-value corresponds to an ${\mathrm{HbO}}_{2}$ increase and an HbR decrease; a negative t-value corresponds to an ${\mathrm{HbO}}_{2}$ decrease and an HbR increase.

**Fig. 8:**
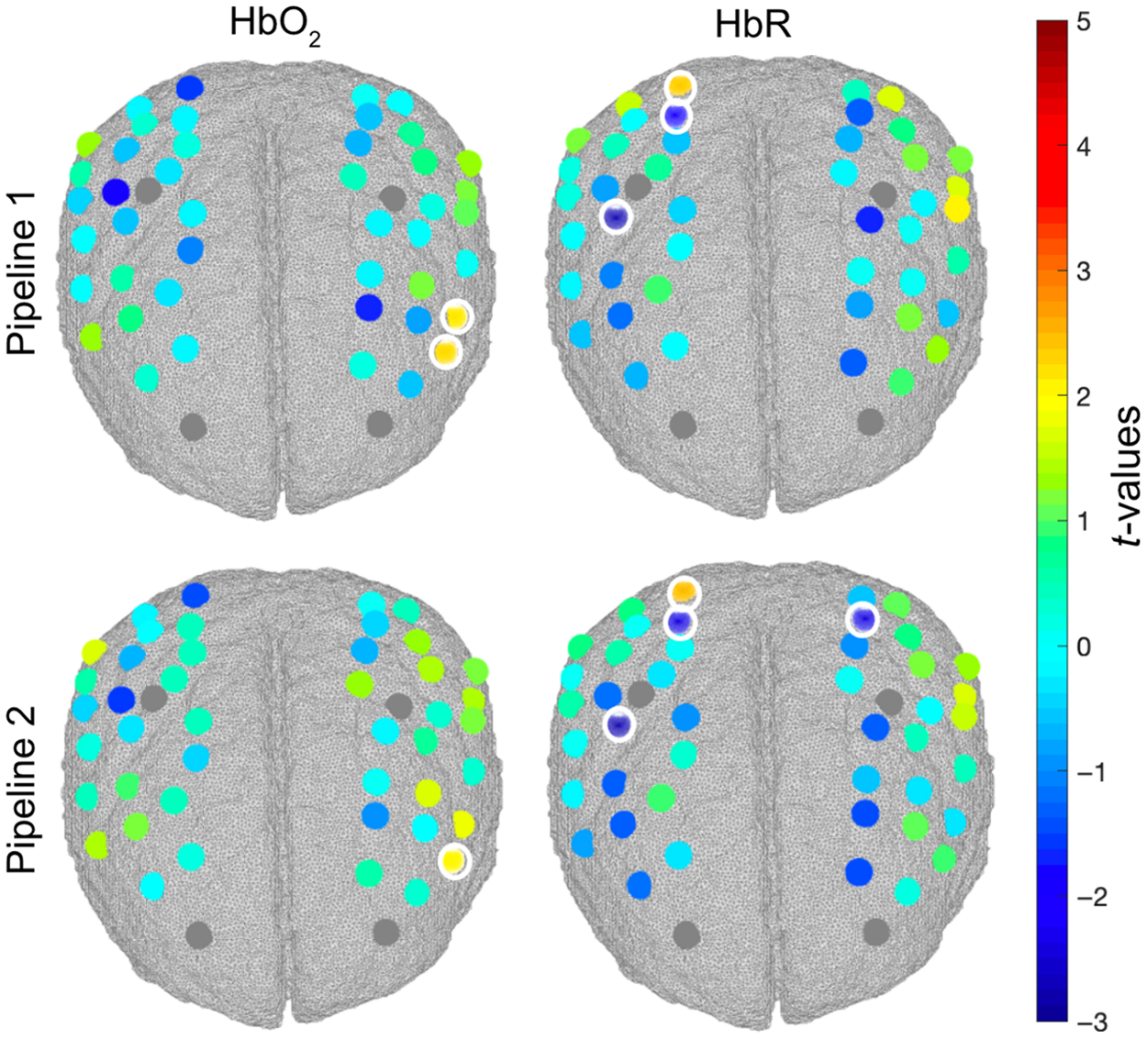
Group level t-value maps for the contrast mixed > go-only for the CB task. Statistically significant channels at p<0.05 are circled in white. Channels surviving FDR correction are marked with asterisks. A positive t-value corresponds to an ${\mathrm{HbO}}_{2}$ increase and an HbR decrease; a negative t-value corresponds to an ${\mathrm{HbO}}_{2}$ decrease and an HbR increase.

To test the effect of the regression of short separation channels on the group-level statistics, we ran channel-wise one-sample t-tests on the group AUCs for our contrast of interest (mixed > go-only) for ${\mathrm{HbO}}_{2}$ and HbR for pipeline 1 and pipeline 2. This was done for both the VR and CB tasks. Group-level t-maps are shown in Figs. 7 and 8 for the VR and CB experiments, respectively, and all statistical results are also reported in Tables S1–S4 in the Supplementary Material.

For the VR task, larger significant changes (p<0.05) in ${\mathrm{HbO}}_{2}$ and HbR can be observed in the mixed blocks compared with the go-only blocks in the channels covering the middle frontal, precentral, postcentral, and inferior frontal gyri directly related to response inhibition, although only channel 9 (precentral gyrus) survives the FDR correction for ${\mathrm{HbO}}_{2}$. The opposite pattern is observed in channels probing the superior parietal gyrus as go-only trials require the continuous execution and planning of movements more than in mixed trials. Looking at the general and uncorrected patterns of brain activity, more widespread activations are found when using pipeline 1. Pipeline 2 yields more localized activations, with 10 significant channels for ${\mathrm{HbO}}_{2}$ (p<0.05) compared with the 15 of pipeline 1. For HbR, the regression of short channels in pipeline 2 increases the statistical significance of two more channels, further maximizing the overlap between ${\mathrm{HbO}}_{2}$ and HbR that better reflects the expected dynamic of brain activity, i.e., increase in ${\mathrm{HbO}}_{2}$ and decrease in HbR.

Similarly for the CB task, we found significant changes (p<0.05) in ${\mathrm{HbO}}_{2}$ and HbR when comparing the mixed condition against the go-only condition in the middle frontal, inferior frontal, and superior parietal gyri, although no channels survive the FDR correction. The regression of short channels results in more localized changes in ${\mathrm{HbO}}_{2}$, with one channel being active versus two of pipeline 1, but the effect is smaller than for the VR task. This suggests that physiological changes might affect brain hemodynamics more in standing (VR case) versus seated (CB case) participants.

**Fig. 9:**
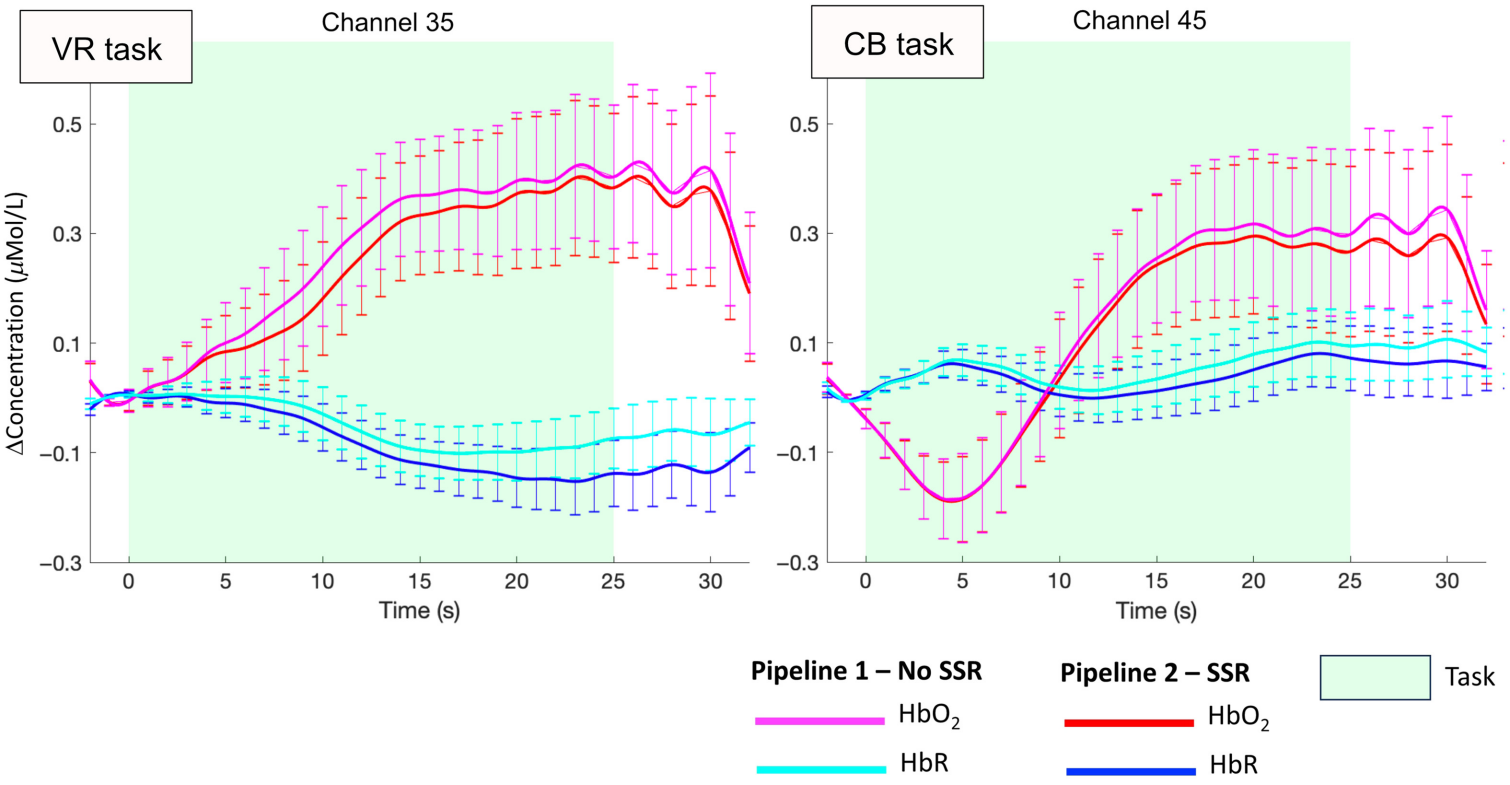
Group-averaged task-evoked responses (mean ± std.err.) for the contrast mixed−go-only. The resulting ${\mathrm{HbO}}_{2}$ and HbR responses from two representative channels are shown both without (magenta and cyan lines) and with (red and blue lines) SSR for the VR and CB tasks. SSR leads to a decrease in ${\mathrm{HbO}}_{2}$ amplitude and an increase in HbR changes.

In general, in both tasks, the superficial signal regression led to a reduction in the amplitude of ${\mathrm{HbO}}_{2}$ and an increase in that of HbR. An example of the resulting group responses to the contrast mixed–go-only is shown in Fig. 9 for two representative channels, for both the VR and CB tasks.

## 4 Discussion

In this work, we investigated for the first time in toddlers and preschoolers if significant hemodynamic and oxygenation changes occur in the extracerebral layers of the head and whether the removal of such components has an impact on the group-level statistics when localizing hemodynamic responses in the cerebral compartment. Specifically, we explored whether the superficial contamination on the cortical brain fNIRS signals was stronger when data were recorded from a developmental sample during a cognitive task while freely moving compared with sitting. To this goal, we collected fNIRS data from 3- to 7-year-olds during a go/no-go task performed while freely moving in an immersive VR CAVE space (VR task) and while sitting in front of a computer (CB task). We applied two different analysis pipelines (Fig. 3) to test the effect of superficial signal regression on the group-level statistics.

Our initial investigations suggested that similar physiological components as adults’ fNIRS data can be observed in children’s fNIRS data as shown by visible Mayer waves in the raw intensity data (Fig. 4) alongside other known components such as heart rate, slow trends, and motion artifacts. If these are not accounted for, they can propagate along the analysis pipeline, possibly ending up affecting the outcome of the statistical analysis. For example, in Fig. 5, we showed that, if appropriate approaches, i.e., band-pass filtering along with SSR such as pipeline 2, are not used, Mayer waves components are still clearly visible in the recovered hemodynamic responses and can affect the amplitude changes of ${\mathrm{HbO}}_{2}$ and HbR, hence leading to false positives and/or false negatives.^9^ In agreement with this, our results showed that group-level t-statistics are either inflated or deflated due to the presence of physiological modulations in the fNIRS signals when pipeline 2 is not used (Tables S1–S4 and Figs. S3–S4 in the Supplementary Material). In fact, we found differences in the group-level t-values across the three pipelines (pipeline 2 versus pipeline 1 with [0.01 0.5] Hz and with [0.01 0.1] Hz band-pass filters) in ${\mathrm{HbO}}_{2}$ and HbR, particularly in the VR task compared with the CB task. The fact that t-values are either inflated or deflated might also further support the possibility that the impact of physiological noise is not homogenous across the head.^43^^,^^44^ In addition, the use of pipeline 2 improved the overlap between the ${\mathrm{HbO}}_{2}$ and HbR statistical results, i.e., the expected pattern in the case of functional brain activity, suggesting its superior efficacy in reducing physiological contaminations.

Similar to previous studies in adults,^11^^,^^21^^,^^43^ we found significant changes in the short separation channels (Fig. 6; Table S5 in the Supplementary Material) both in the VR task and in the CB task, indicating that physiological contamination can occur not only when children are standing or moving around but also when they are performing standard computer-based tasks. Generally, we found that ${\mathrm{HbO}}_{2}$ increases and HbR decreases during the CB task and that ${\mathrm{HbO}}_{2}$ decreases and HbR increases in the VR task in the SSCs. We hypothesize that the superficial decreases in ${\mathrm{HbO}}_{2}$ and increases in HbR trends in the VR task might be due to respiration-induced changes in physiological variables such as in the ${\mathrm{PaCO}}_{2}$. Previous studies have reported this inverted pattern in the case of hypocapnia.^45^^,^^46^ Changes in respiratory patterns can be expected in the case of dynamic tasks, in which the participant is standing and performing larger body movements (e.g., walk to reach a bubble or move the whole arm to pop the bubble) than a standard computer-based task in which just a key press is required. Further investigation with physiological measurements (Systemic Physiology Augmented fNIRS (SPA-fNIRS),^7^^,^^47^ e.g., by measuring respiration or ${\mathrm{PaCO}}_{2}$ alongside fNIRS, is needed to further confirm this hypothesis.

Such superficial changes seem to be heterogeneous across the scalp as previously reported in adults,^43^^,^^44^ showing different trends across the four SSCs (Fig. 6). This is also reflected at the group level as, when incorporating SSR in pipeline 2, t-values either increase or decrease across channels compared with pipeline 1 with no SSR (Tables S1–S4 in the Supplementary Material). Hence, the use of multiple short separation channels and regressing them out locally can be highly beneficial in this population as much as it is in adults. In this framework, previous studies^10^ have proposed removing a global component only or in addition to local SSR regression;^20^ the global component can be obtained by averaging all SSCs or computing the first two principal components of the combined SSCs^21^ or in addition to local SSR regression.^20^ However, we believe that such approaches would perform better when a larger head coverage or high-density measurements with several SSCs are available. These configurations would allow for a more accurate estimation of a global component, i.e., signal changes that affect the whole brain or large regions, which might not be estimated accurately with our sparse and low coverage array with up to four SSCs. In fact, when using a global SSR approach on our data by averaging all SSCs, we obtained very similar results to local SSR, suggesting that the SSCs average might not reflect a truly global component (Tables S1–S4 and Figs. S3–S4 in the Supplementary Material). Therefore, in this study, we regressed SSCs locally rather than globally considering the one maximally correlated to each LSC.

Our exploratory analyses confirmed that the go/no-go task elicited significant brain activity in the expected regions of interest within dlPFC,^48^^,^^49^ with larger changes in brain hemodynamics in the mixed blocks compared with the go-only blocks per effect of response inhibition. The opposite pattern (mixed < go-only) was found particularly for the VR task in the more posterior regions covering the motor/superior parietal areas^50^ and mostly in the left hemisphere (e.g., channels 25, 26, and 27), contralateral to the arm movement. These regions are involved in motor execution and planning and are expected to show more sustained and higher activity during go-only blocks for which a continuous response/movement is required than in mixed blocks for which movements need to be intermittently suppressed.^51^^,^^52^ This is especially true for the VR task in which participants need to perform a larger movement with the right arm instead of a simple button press such as in the CB task. Results also revealed that the regression of short separation channels has an effect on the group-level statistical maps for both the VR and CB tasks (Figs. 7 and 8) and across both more anterior and posterior brain regions. In particular, pipeline 2 (with SSR) led to more localized activation patterns compared with pipeline 1 (without SSR) for ${\mathrm{HbO}}_{2}$ as reflected by the smaller number of active channels. The improvement in spatial localization has also been reported by previous fNIRS studies on adults (e.g., Refs. 21 and 53). SSR also increased the statistical significance of HbR (Fig. 7), further improving the detection of brain activity (i.e., increase in ${\mathrm{HbO}}_{2}$ and decrease in HbR). The increase in spatial localization per effect of SSR was more pronounced for the VR task (Fig. 7) than for the CB task (Fig. 8), suggesting that, in agreement with our hypothesis, systemic interferences might have a larger impact when fNIRS data are recorded in more physically active conditions.^15^^,^^17^ Nevertheless, SSR influences group-level results also for the CB experiment, though to a lesser extent (Fig. 8). Therefore, we also recommend the use of short separation channels in instances in which fNIRS data are recorded from children in typical non-mobile neuroimaging contexts. Across both tasks, most of the changes post-SSR can be observed in the ${\mathrm{HbO}}_{2}$ signal, in agreement with previous work demonstrating that oxyhemoglobin is more sensitive to physiological interferences than HbR.^29^

In summary, our preliminary work represents the first report of the occurrence and influence of scalp blood flow changes on the estimation of brain activity through fNIRS in toddlers and preschoolers. As such, it provides the initial evidence that (1) significant changes in scalp hemodynamics can occur in toddlers and preschoolers, (2) extracerebral changes seem to be stronger when children perform cognitive tasks while moving versus when they are sitting, (3) SSR can influence the outcome of group-level statistics by improving spatial localization of hemodynamic responses, and (4) short separation channels can be highly beneficial in 3- to 7-year-olds both in mobile and non-mobile experimental setups. However, limitations to this work should be considered. First, the sample size was not ideal for all channels (Table 1). This depended on whether the optodes were probing brain activity on hairy regions, which worsened the optical coupling in some participants; when collecting data on young children, coupling optimization procedures cannot always take place or need to be done very quickly to avoid kids becoming uncompliant. In addition, the VR and the CB tasks involved different body movements and changes in optical coupling from one task to the other, and re-optimization procedures could not always take place; for example, some children moved their heads more during the CB task than the VR task. Collecting data from a larger cohort of children could help in mitigating these issues in future studies. It is worth mentioning that, here, we used a source–detector separation of 1 cm for SSCs, being constrained by the physical dimension of the optodes of the fNIRS systems used in the current setup. Based on our preliminary sensitivity analysis (Sec. 1 in the Supplementary Material), SSCs at 1 cm may still include some contribution from the deeper brain areas, particularly in the younger age range of our sample. Therefore, our results might have been affected by the removal of part of brain hemodynamic changes in some participants. Future studies should consider reducing the source–detector separations for short channels to at least 0.8 mm to minimize their sensitivity to the brain when collecting fNIRS data on toddlers. If allowed by the fNIRS hardware, whole-head or high-density measures can also help in this regard to further reduce scalp contamination by adding the removal of global systemic components.^20^^,^^21^ In addition, we have not used additional monitors of systemic physiology (e.g., ECG, respiration, and blood pressure) that could have helped to better assess the presence of significant task-related physiological changes and further improve the robustness of fNIRS signals. This was partly related to practical challenges in using several pieces of equipment on young children as in our study they were already wearing the fNIRS cap, a motion tracking glove, and head-mounted shutter glasses/head tracking (Fig. 1). Nevertheless, previous work has shown that an SPA-fNIRS approach^7^^,^^47^ can be highly beneficial in denoising the fNIRS signals from systemic interferences. This would be worth exploring in children in future studies. Alongside physiological monitors, in our future work, we plan on using signals coming from motion tracking, such as head movements, as additional nuisance regressors. In fact, von Lühmannet al.^54^ showed that combining short separation channels with accelerometer signals on the head can significantly improve the recovery of the hemodynamic responses. Finally, we chose a source–detector separation of 1 cm due to the physical constraints of the optodes size; even though young children seem to have a similar sensitivity profile to adults^55^ and similar head sizes, it is possible that part of the signal of the short separation channels is of cortical origin (Sec. 1 in the Supplementary Material); therefore, shorter source–detector separations might be better suited for SSR in children^23^ if the hardware allows.

## 5 Conclusion and Methodological Recommendations

In this study, we have provided the first evidence that superficial signal contamination of fNIRS data also occurs in younger children. Interestingly, our results indicate that children aged 3 to 7 years are more similar to adult populations than infants in terms of non-neural components in the fNIRS signals. In fact, previous work from Emberson et al.^23^ showed that the removal of superficial vasculature signals did not have a significant impact on group-level statistics in 6-month-old infants. Nonetheless, it is unclear whether other, more active or physiologically arousing paradigms might show greater contributions of superficial vasculature signals even in infants. The assumption that the fNIRS signal is a mixture of components of both neuronal and non-neuronal origin^9^ seems to be valid in children as well and can potentially lead to false positives and false negatives at the statistical inference stage. Therefore, we suggest that removing the contribution of superficial signals to the long fNIRS channels is an important step in the analysis pipeline of children’s data, especially when fNIRS signals are recorded in experimental contexts that elicit stronger physiological changes (e.g., standing, walking, and full body movements). Future work will be important to further explore how other cognitive tasks and experimental settings modulate the impact of systemic interferences in children as well as the feasibility of SPA-fNIRS in these populations.

**Fig. 10:**
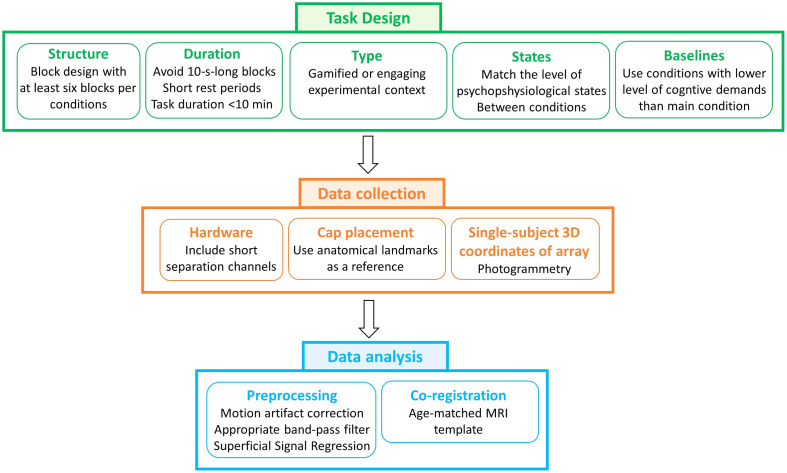
Summary of methodological considerations for robust children neuroimaging experiments.

Task design, array co-registration, and the preprocessing pipeline were optimized for children to maximize the robustness of the results. Based on our results, below, we provide preliminary recommendations as a first step to advance the field of children’s fNIRS neuroimaging. Figure 10 shows some of the steps that we recommend to enhance the robustness of the results of fNIRS data recorded on children and particularly in ecological settings.

### 5.1 Methodological Considerations for Task Design

Tasks were designed to meet different requirements.

First, it is well known that pediatric neuroimaging can present several challenges due to their compliance with neuroimaging experimental requirements, particularly in preschoolers.^56^ The CAVE represents a much more engaging setting for children, increasing their compliance together with fNIRS being a child-friendly technique.^57^ Hence, longer tasks with a larger number of trials that increase the statistical power of the study may be potentially suitable. However, in this work, a conventional computerized task was needed for the validation of the novel VR naturalistic task and to evaluate the differences in physiological interferences between the two. To maximize children’s cooperation and participant retention in two experiments, both tasks were kept to a relatively limited duration of 6 to 8 min each while ensuring a good number of trials. In particular, six blocks per condition were used to ensure that, in the case of performance- or attention-based block exclusion, the chances of ending up with at least three blocks per condition were high (note: a threshold of a minimum of three blocks for participants’ inclusion is often used in fNIRS developmental research^58^).

Second, to maximize the signal-to-noise ratio of the data, we chose block-designed tasks rather than an event-related design as the latter requires a larger number of events and usually elicits lower contrast hemodynamic responses above the noise level.^59^ Along these lines, block designs typically include repeated rest periods to allow the slow hemodynamic response to go back to baseline; however, it has been suggested that it is not fully appropriate to contrast the task to the rest blocks as it is unknown what the participant is really doing during that rest time, and hence it might not be a realistic baseline. It is instead recommended to use low-level control conditions for experimental contrasts.^59^^,^^60^ Therefore, our task was designed such that mixed blocks were contrasted to the lower-level go-only blocks. Short rest periods were added in between to avoid the overlap of the various hemodynamic responses but were not used for comparing conditions.

Finally, to help minimize the impact of physiological interferences especially in the VR task, we avoided 10-s-long blocks,^9^ and the conditions (go-only and mixed) were planned in a way to have the same level of physical activity. In this way, when contrasting the mixed blocks versus go-only, some of the confounding effects may cancel out.^60^ In the VR task, both conditions required the participants to stand and to move the arm in the same way; in the CB task, a button press was always needed.

### 5.2 Methodological Considerations for Data Collection

To take into account task-evoked extracerebral changes, we suggest adding short separation channels to the fNIRS array, even if the experiment is performed while sitting. We recommend including at least one SSC although it has been previously demonstrated that scalp interference is not homogenous and, based on hardware availability, a larger number of SSC scattered around the head is advised.^61^

To account for between-subjects variability in the location of the channels, the co-registration of the fNIRS array onto a common brain template is typically recommended, in addition to ensuring a reliable cap placement using anatomical landmarks as a reference. 3D magnetic digitizers are generally used in fNIRS (and EEG) studies to gather the 3D coordinates of the optodes and landmarks. However, these require the participant to stay still for several minutes and are also susceptible to electromagnetic interference from the environment.^60^ Therefore, it may not be feasible to have children stay still for long periods of time. To overcome this barrier, here, we used a photogrammetry-based method (STORM-Net^35^ but others are available^62^^–^^64^) that is compatible with children’s movements and does not require extra hardware except a smartphone camera.

### 5.3 Methodological Considerations for Data Analysis

In terms of the preprocessing of fNIRS data, as demonstrated by previous studies, we recommend including motion artifact correction rather than trial rejection in the preprocessing pipeline;^65^^–^^68^ this ensures that a larger number of trials can be retained, which is particularly important when working with developmental populations in which the number of good quality blocks might be limited. Following the procedure described in Ref. 8, we recommend choosing a filter that is appropriate for the timings of the task and, when possible, using a narrower low pass filter with a cutoff frequency close to 0.1 Hz (the Mayer waves fundamental frequency) to help attenuate its impact. Here, for example, we used a Butterworth band-pass filter within the range [0.01 0.1] Hz. To improve the localization of functional brain activity by minimizing the effect of systemic interferences, the regression of short separation channels from the long separation channels seems to be an important step also in young children, and hence, we advise incorporating it within the analysis pipeline. When allowed by the fNIRS instrumentation, the source–detector separation for short channels should also be <1  cm to minimize sensitivity to the brain. Finally, to further improve the robustness of the inferences about the task-evoked hemodynamic activity, it is important to know the underlying anatomical location of the fNIRS channel. This allows for the grouping of anatomically and functionally homogenous regions in the group-level analysis and can be achieved by co-registering the 3D coordinates of the optodes/channels onto a common brain template. Although co-registering the channels onto the subject-specific structural MRI can better capture the individual variation in cortical anatomy,^57^ these are rarely available, especially for children; therefore, we recommend using an averaged age-matched template that can be found available on open access repositories. For example, here, we have used the Neurodevelopmental MRI Database of the University of South Carolina.^32^

## Supplementary Material



## Data Availability

The data supporting the results of this paper can be made available upon reasonable request to the corresponding author through a formal data sharing and project affiliation agreement.
