# Supplementary material for: Ecological functional near-infrared spectroscopy in mobile children: using short separation channels to correct for systemic contamination during naturalistic neuroimaging
Source: Neurophotonics. 2024 Oct 8;11(4):045004. doi: 10.1117/1.NPh.11.4.045004 (PMC11460616; doi:10.1117/1.NPh.11.4.045004)
Supplement: Supplementary file 1 [file NPh_011_045004_SD001.pdf]

1. Preliminary sensitivity analysis

A preliminary analysis on the sensitivity distribution of our fNIRS array (Figure 2 in the main manuscript) was performed to investigate the age-related differences in channels' brain sensitivity and gather preliminary information on the optimum source-detector separation for short channels for toddlers and preschoolers that minimizes the signal contribution coming from the brain.

To this goal, we first built two age-appropriate head models for toddlers and preschoolers. These were created using the 3-years old and 5-years old MRI templates from the the Neurodevelopmental MRI Database of the University of South Carolina (https://www.nitrc.org/projects/neurodevdata). The MRI templates were segmented using the FMRIB Software Library (FSL [35]) and a volumetric four-layer mask (extra-cerebral tissue (ECT), cerebrospinal fluid (CSF), grey matter (GM), white matter (WM); [65, 66]) was created using routines from the DOT-HUB toolbox (https://github.com/DOT-HUB). A tetrahedral volumetric mesh and a GM surface mesh were generated; the optodes' coordinates were co-registered onto the scalp mesh first via affine transformation. A forward model at each wavelength was produced using the DOT-HUB toolbox by modelling near-infrared light transport in tissue through TOAST++ [67] (http://web4.cs.ucl.ac.uk/research/vis/toast/) and the finite element method. The Jacobian or sensitivity matrix was computed in a fine regular grid with size 30 x 30 x 30 voxels [66] and mapped to the volumetric mesh and then to the GM mesh. A binary sensitivity mask was then created in the GM space by setting to 1 all the nodes that exhibited a sensitivity value above the 5% of the maximum value of the normalized Jacobian [66]. This procedure was applied to both the 3-year-old and 5-year-old head models considering a channel layout that includes long separation channels (LSCs) at 2.5 cm source-detector separation and short separation channels (SSCs) at 1 cm. In addition, source-detector distances of 0.8 cm and 0.6 cm for SSCs were further explored. This was done to investigate which short source-detector separation is the least sensitive to the brain and hence is the most appropriate for superficial signal regression for toddlers and preschoolers.

Supplementary Figure 1 A presents the sensitivity maps and the binary masks for our full arrays including LSCs at 2.5 cm and SSCs at 1 cm for the age group 3 years old (left) and 5 years old (right), showing that similar sensitivity profiles and brain regions are probed in the two groups. A similar sensitivity to the brain is achieved with a 2.5 cm LSC in both the 3- and 5-years old groups (Supplementary Figure 1 B, top panels); however, the four SSCs of our array at 1 cm distance are also sensitive to the brain in 3-years olds and in a very minor extent in 5-years olds (Supplementary Figure 1 B, bottom panels).

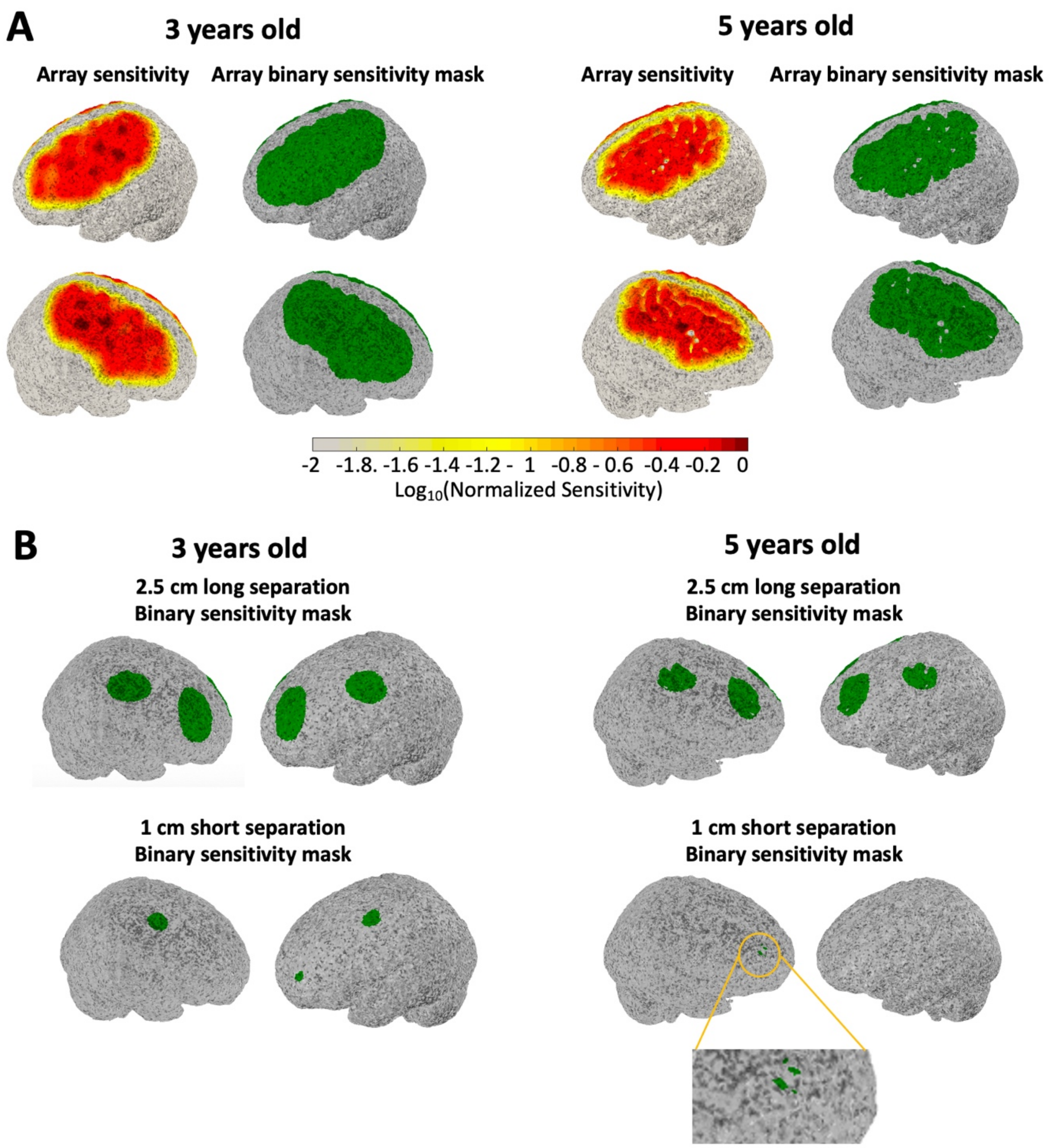

Supplementary Figure 1. Array sensitivity profiles and binary sensitivity maps for 3- and 5-years old head models. Panel A shows the sensitivity distributions and corresponding binary sensitivity maps across the whole 48-channels array, including long separation channels (LSCs) at 2.5 cm and short separation channels (SSCs) at 1 cm. Panel B shows the brain binary sensitivity maps of four representative LSCs (channels 1, 12, 34, 37) and for the four SSCs (channels 3, 13, 17, 26).

Reducing the source-detector distance to 0.8 cm seems to eliminate SSC sensitivity to the brain in the older age group (Supplementary Figure 2, right panels) but still leaves some very small clusters in the younger age group (Supplementary Figure 2, top left panels). These disappear if a source-detector separation of 0.6 cm is used (Supplementary Figure 2, bottom left panels).

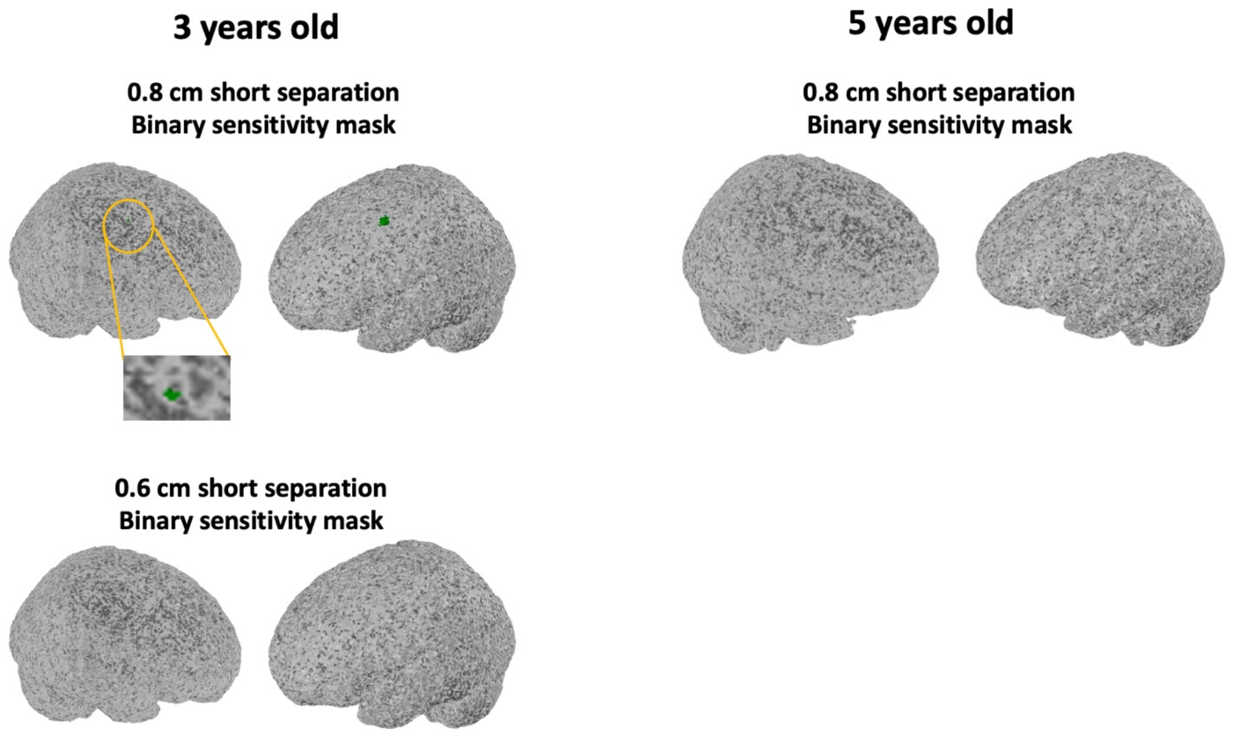

Supplementary Figure 2. Binary sensitivity maps for 3- and 5-years old head models at different source-detector separations (0.8 cm and 0.6 cm).

Even though these results are preliminary and further systematic investigations are needed, they provide initial evidence that a source-detector separation of 0.8 cm can offer a good compromise in terms of minimum brain sensitivity of SSCs across our whole age group (3-to-7 years old), similarly to what (Brigadoi & Cooper, 2015) recommended for adults.

2. Group-level statistical analysis results for the contrast of interest Mixed>Go-only across different pipelines

Supplementary Table 1. Group level t-values maps for the contrast Mixed>Go-only for HbO_2 for the VR task. Results are reported for pipeline 1 with a band-pass filter with cut-off frequencies [0.01 0.5 Hz] and [0.01 0.1] Hz, pipeline 2 regressing out the short separation channel with the highest correlation (SSR correlation) and pipeline 2 regressing out the average of all short separation channels (SSR average). Single asterisks indicate significant channels at $p<0.05$ uncorrected; double asterisks indicate significant channels surviving false discovery rate multiple comparison correction at $p<0.05$. A positive t-value corresponds to a larger HbO_2 increase in the Mixed blocks compared to the Go-only blocks.

HbO_2		Pipeline 1 with [0.01 0.5] Hz filter		Pipeline 1 with [0.01 0.1] Hz filter		Pipeline 2 SSR correlation		Pipeline 2 SSR average	
	**df**	**t-value**	**p-value**	**t-value**	**p-value**	**t-value**	**p-value**	**t-value**	**p-value**
**Ch 1**	27	2.05*	0.05	1.86	0.07	1.77	0.09	1.82	0.08
**Ch 2**	28	2.31*	0.03	2.27*	0.03	2.03	0.05	2.06*	0.05
**Ch 3**	-	-	-	-	-	-	-	-	-
**Ch 4**	19	1.54	0.14	1.37	0.19	1.22	0.24	1.09	0.29
**Ch 5**	18	0.76	0.46	0.66	0.52	0.42	0.68	0.23	0.82
**Ch 6**	29	2.71*	0.01	2.72*	0.01	2.48*	0.02	2.68*	0.01
**Ch 7**	25	3.11*	0.005	3.00*	0.01	2.72*	0.01	2.88*	0.01
**Ch 8**	26	0.56	0.58	0.50	0.62	0.68	0.50	0.52	0.61
**Ch 9**	24	4.85**	0.0001	5.01**	0.00004	4.93**	0.00005	4.69**	0.00009
**Ch 10**	16	0.13	0.90	0.18	0.86	0.20	0.84	0.25	0.81
**Ch 11**	26	2.33*	0.03	2.33*	0.03	1.80	0.08	2.02	0.05
**Ch 12**	24	0.61	0.55	0.61	0.55	0.22	0.83	0.26	0.80
**Ch 13**	17	1.23	0.24	1.37	0.19	1.09	0.29	1.14	0.27
**Ch 14**	-	-	-	-	-	-	-	-	-
**Ch 15**	27	3.15*	0.004	3.12*	0.004	2.51*	0.02	2.70*	0.01
**Ch 16**	25	1.00	0.33	1.05	0.30	0.60	0.56	0.84	0.41
**Ch 17**	21	-0.33	0.74	-0.16	0.88	-0.54	0.59	-0.32	0.75
**Ch 18**	13	1.28	0.22	1.45	0.17	1.21	0.25	1.28	0.22
**Ch 19**	20	1.07	0.30	1.10	0.29	0.85	0.41	0.87	0.39
**Ch 20**	13	1.11	0.29	1.06	0.31	0.58	0.57	0.79	0.44
**Ch 21**	23	2.24*	0.03	2.45*	0.02	1.93	0.07	2.19*	0.04
**Ch 22**	25	2.66*	0.01	2.64*	0.01	2.36*	0.03	2.35*	0.03
**Ch 23**	22	-1.21	0.24	-1.31	0.21	-1.31	0.20	-1.53	0.14
**Ch 24**	18	-1.84	0.08	-2.28*	0.03	-2.98*	0.01	-2.75*	0.01
**Ch 25**	14	-2.13	0.05	-2.22*	0.04	-2.30*	0.04	-2.32*	0.04
**Ch 26**	23	-1.68	0.11	-1.69	0.10	-1.57	0.13	-1.43	0.17

**Ch 27**	10	-1.63	0.13	-1.68	0.12	-2.37*	0.04	-2.11	0.06
**Ch 28**	13	0.04	0.97	0.11	0.91	-0.11	0.91	0.02	0.99
**Ch 29**	15	-2.28*	0.04	-2.14*	0.05	-2.11	0.05	-2.34*	0.03
**Ch 30**	12	-0.22	0.83	0.00	1.00	-0.38	0.71	-0.43	0.68
**Ch 31**	20	-0.80	0.43	-0.70	0.49	-0.71	0.49	-0.56	0.58
**Ch 32**	22	0.29	0.78	0.36	0.72	0.27	0.79	0.42	0.68
**Ch 33**	12	1.12	0.29	1.00	0.34	0.51	0.62	0.35	0.73
**Ch 34**	17	2.41*	0.03	2.18*	0.04	2.01	0.06	2.01	0.06
**Ch 35**	21	3.22*	0.004	2.88*	0.01	2.48*	0.02	2.57*	0.02
**Ch 36**	12	1.85	0.09	1.79	0.10	1.10	0.29	1.19	0.26
**Ch 37**	-	-	-	-	-	-	-	-	-
**Ch 38**	27	1.73	0.10	1.83	0.08	1.66	0.11	1.87	0.07
**Ch 39**	24	0.97	0.34	0.83	0.42	0.57	0.57	0.91	0.37
**Ch 40**	27	1.68	0.10	1.76	0.09	1.33	0.19	1.32	0.20
**Ch 41**	18	2.23*	0.04	2.26*	0.04	1.93	0.07	1.77	0.09
**Ch 42**	27	0.06	0.95	0.16	0.87	-0.42	0.68	-0.15	0.88
**Ch 43**	22	1.90	0.07	1.59	0.13	1.42	0.17	1.07	0.29
**Ch 44**	19	1.50	0.15	1.45	0.16	1.18	0.25	1.54	0.14
**Ch 45**	21	1.97	0.06	2.11*	0.05	2.46*	0.02	2.37*	0.03
**Ch 46**	27	0.90	0.38	0.89	0.38	0.84	0.41	0.56	0.58
**Ch 47**	24	1.18	0.25	0.97	0.34	1.32	0.20	0.50	0.62
**Ch 48**	-	-	-	-	-	-	-	-	-

Supplementary Table 2. Group level t-values maps for the contrast Mixed>Go-only for HbR for the VR task. Results are reported for pipeline 1 with a band-pass filter with cut-off frequencies [0.01 0.5 Hz] and [0.01 0.1] Hz, pipeline 2 regressing out the short separation channel with the highest correlation (SSR correlation) and pipeline 2 regressing out the average of all short separation channels (SSR average). Single asterisks indicate significant channels at p<0.05 uncorrected; double asterisks indicate significant channels surviving false discovery rate multiple comparison correction at p<0.05. A positive t-value corresponds to a larger HbR decrease in the Mixed blocks compared to the Go-only blocks.

HbR		Pipeline 1 with [0.01 0.5] Hz filter		Pipeline 1 with [0.01 0.1] Hz filter		Pipeline 2 SSR correlation		Pipeline 2 SSR average	
	**df**	**t-value**	**p-value**	**t-value**	**p-value**	**t-value**	**p-value**	**t-value**	**p-value**
**Ch 1**	27	-0.96	0.34	-0.83	0.42	-0.99	0.33	-1.32	0.20
**Ch 2**	28	-2.82*	0.01	-2.56*	0.02	-2.17*	0.04	-2.17*	0.04
**Ch 3**	-	-	-	-	-	-	-	-	-
**Ch 4**	19	0.01	0.99	-0.10	0.92	-0.04	0.96	-0.56	0.58
**Ch 5**	18	0.33	0.74	0.29	0.78	0.81	0.43	0.37	0.72
**Ch 6**	29	-1.48	0.15	-1.50	0.15	-1.23	0.23	-1.50	0.14
**Ch 7**	25	-1.75	0.09	-1.76	0.09	-1.55	0.13	-1.77	0.09
**Ch 8**	26	-0.39	0.70	-0.26	0.80	-0.32	0.75	-0.25	0.80
**Ch 9**	24	0.03	0.98	0.10	0.92	-0.32	0.75	0.04	0.97
**Ch 10**	16	-0.52	0.61	-0.31	0.76	0.60	0.55	0.16	0.88
**Ch 11**	26	-1.26	0.22	-1.25	0.22	-0.94	0.36	-0.97	0.34
**Ch 12**	24	-0.10	0.92	-0.12	0.91	0.02	0.98	-0.10	0.92
**Ch 13**	17	0.09	0.93	0.07	0.95	0.51	0.61	0.05	0.96
**Ch 14**	-	-	-	-	-	-	-	-	-
**Ch 15**	27	0.03	0.97	-0.07	0.95	-0.19	0.85	0.03	0.98
**Ch 16**	25	-0.13	0.90	-0.04	0.97	0.04	0.97	0.16	0.88
**Ch 17**	21	-1.39	0.18	-1.55	0.14	-1.67	0.11	-1.84	0.08
**Ch 18**	13	-0.03	0.97	-0.13	0.90	-0.22	0.83	-0.36	0.73
**Ch 19**	20	-0.53	0.60	-0.52	0.61	-0.67	0.51	-0.42	0.68
**Ch 20**	13	0.02	0.99	0.19	0.85	-0.03	0.98	0.06	0.96
**Ch 21**	23	0.89	0.38	0.90	0.38	1.27	0.22	1.13	0.27
**Ch 22**	25	1.70	0.10	1.75	0.09	1.70	0.10	1.89	0.07
**Ch 23**	22	0.62	0.54	0.74	0.47	0.22	0.82	0.84	0.41
**Ch 24**	18	-0.63	0.54	-0.70	0.49	-0.87	0.40	-0.28	0.79
**Ch 25**	14	3.12*	0.01	3.10*	0.01	3.36*	0.005	3.70*	0.002
**Ch 26**	23	2.44*	0.02	2.58*	0.02	2.64*	0.01	2.36*	0.03
**Ch 27**	10	4.04*	0.002	4.17*	0.002	2.62*	0.03	3.45*	0.01
**Ch 28**	13	0.24	0.81	0.02	0.98	-0.35	0.73	-0.12	0.90
**Ch 29**	15	1.74	0.10	1.76	0.10	1.85	0.08	1.85	0.08
**Ch 30**	12	0.23	0.82	0.20	0.85	-0.07	0.95	0.19	0.86

**Ch 31**	20	1.60	0.12	1.57	0.13	1.66	0.11	1.45	0.16
**Ch 32**	22	0.11	0.92	0.33	0.75	-0.15	0.88	-0.17	0.87
**Ch 33**	12	0.11	0.92	0.14	0.89	-0.29	0.78	0.06	0.95
**Ch 34**	17	-2.10	0.05	-1.92	0.07	-2.18*	0.04	-2.08	0.05
**Ch 35**	21	-1.77	0.09	-1.61	0.12	-2.33*	0.03	-1.62	0.12
**Ch 36**	12	0.41	0.69	0.44	0.67	0.32	0.75	0.34	0.74
**Ch 37**	-	-	-	-	-	-	-	-	-
**Ch 38**	27	-2.11*	0.04	-1.83	0.08	-1.91	0.07	-1.81	0.08
**Ch 39**	24	-0.92	0.37	-0.90	0.37	-0.84	0.41	-1.15	0.26
**Ch 40**	27	-1.50	0.15	-1.48	0.15	-1.65	0.11	-1.45	0.16
**Ch 41**	18	0.16	0.87	0.37	0.72	0.57	0.58	0.30	0.77
**Ch 42**	27	-0.54	0.59	-0.71	0.49	-0.42	0.68	-0.54	0.59
**Ch 43**	22	1.05	0.31	0.97	0.34	0.91	0.37	1.09	0.29
**Ch 44**	19	1.49	0.15	1.46	0.16	1.50	0.15	1.32	0.20
**Ch 45**	21	0.28	0.78	0.33	0.74	-0.21	0.83	-0.12	0.91
**Ch 46**	27	-0.23	0.82	-0.15	0.88	-0.62	0.54	-0.38	0.71
**Ch 47**	24	-0.08	0.94	0.00	1.00	0.07	0.94	-0.19	0.85
**Ch 48**	-	-	-	-	-	-	-	-	-

Supplementary Table 3. Group level t-values maps for the contrast Mixed>Go-only for HbO_2 for the CB task. Results are reported for pipeline 1 with a band-pass filter with cut-off frequencies [0.01 0.5 Hz] and [0.01 0.1] Hz, pipeline 2 regressing out the short separation channel with the highest correlation (SSR correlation) and pipeline 2 regressing out the average of all short separation channels (SSR average). Single asterisks indicate significant channels at $p<0.05$ uncorrected; double asterisks indicate significant channels surviving false discovery rate multiple comparison correction at $p<0.05$. A positive t-value corresponds to a larger HbO_2 increase in the Mixed blocks compared to the Go-only blocks.

HbO_2		Pipeline 1 with [0.01 0.5] Hz filter		Pipeline 1 with [0.01 0.1] Hz filter		Pipeline 2 SSR correlation		Pipeline 2 SSR average	
	df	t-value	p-value	t-value	p-value	t-value	p-value	t-value	p-value
**Ch 1**	23	-0.37	0.71	-0.20	0.84	0.19	0.85	0.50	0.62
**Ch 2**	27	0.23	0.82	0.29	0.78	-0.10	0.92	1.21	0.23
**Ch 3**	-	-	-	-	-	-	-	-	-
**Ch 4**	19	-0.29	0.77	-0.34	0.74	0.39	0.70	-0.01	0.99
**Ch 5**	12	-1.00	0.34	-1.09	0.30	-0.46	0.65	-0.77	0.46
**Ch 6**	26	0.84	0.41	0.76	0.45	1.13	0.27	1.22	0.23
**Ch 7**	24	0.44	0.66	0.55	0.59	0.99	0.33	1.32	0.20
**Ch 8**	29	1.39	0.18	1.37	0.18	1.48	0.15	1.86	0.07
**Ch 9**	25	-0.22	0.83	-0.13	0.90	0.48	0.63	0.74	0.47
**Ch 10**	14	-0.18	0.86	-0.21	0.84	0.41	0.69	0.09	0.93
**Ch 11**	22	-0.60	0.56	-0.53	0.60	-0.30	0.77	-0.31	0.76
**Ch 12**	21	-1.79	0.09	-1.81	0.08	-1.57	0.13	-1.37	0.19
**Ch 13**	15	-0.18	0.86	-0.34	0.74	0.43	0.67	0.42	0.68
**Ch 14**	-	-	-	-	-	-	-	-	-
**Ch 15**	26	-0.28	0.78	-0.13	0.90	0.18	0.86	0.25	0.80
**Ch 16**	25	-0.63	0.54	-0.52	0.61	-0.41	0.69	-0.26	0.80
**Ch 17**	21	-0.76	0.46	-0.64	0.53	-0.75	0.46	-0.67	0.51
**Ch 18**	14	0.07	0.94	0.17	0.87	0.47	0.64	0.52	0.61
**Ch 19**	19	0.19	0.85	0.26	0.80	-0.11	0.91	0.20	0.85
**Ch 20**	15	-0.25	0.80	-0.15	0.88	0.46	0.65	0.26	0.80
**Ch 21**	22	0.49	0.63	0.51	0.62	0.53	0.60	0.74	0.47
**Ch 22**	24	1.33	0.19	1.36	0.19	1.70	0.10	1.57	0.13
**Ch 23**	24	-0.36	0.72	-0.19	0.85	-0.26	0.80	0.14	0.89
**Ch 24**	20	-1.43	0.17	-1.54	0.14	-1.43	0.17	-1.52	0.14
**Ch 25**	16	0.01	0.99	0.05	0.96	0.01	0.99	0.15	0.88
**Ch 26**	26	0.04	0.97	-0.03	0.98	0.37	0.72	0.02	0.99
**Ch 27**	15	-0.68	0.50	-0.64	0.53	-0.50	0.63	-0.43	0.67
**Ch 28**	14	-0.76	0.46	-0.70	0.50	-0.67	0.51	-0.52	0.61
**Ch 29**	19	0.78	0.44	0.71	0.49	1.31	0.21	1.38	0.18
**Ch 30**	15	0.95	0.36	0.80	0.44	1.51	0.15	1.62	0.13

**Ch 31**	21	1.25	0.23	1.26	0.22	1.15	0.26	1.17	0.26
**Ch 32**	21	1.13	0.27	1.24	0.23	1.50	0.15	1.43	0.17
**Ch 33**	13	0.45	0.66	0.40	0.70	1.37	0.19	1.37	0.19
**Ch 34**	16	0.21	0.84	0.12	0.90	0.25	0.81	0.45	0.66
**Ch 35**	20	0.03	0.98	-0.02	0.98	0.65	0.52	1.05	0.31
**Ch 36**	10	-0.14	0.89	-0.19	0.85	-0.15	0.89	0.30	0.77
**Ch 37**	-	-	-	-	-	-	-	-	-
**Ch 38**	25	1.08	0.29	1.08	0.29	1.20	0.24	1.03	0.31
**Ch 39**	25	0.00	1.00	-0.01	0.99	0.31	0.76	0.24	0.81
**Ch 40**	26	1.35	0.19	1.14	0.27	1.69	0.10	2.11*	0.04
**Ch 41**	14	-0.10	0.92	-0.20	0.84	0.01	0.99	0.09	0.93
**Ch 42**	25	-0.70	0.49	-0.90	0.38	-0.09	0.93	0.15	0.88
**Ch 43**	17	-1.72	0.10	-1.79	0.09	-0.96	0.35	-1.09	0.29
**Ch 44**	25	2.00	0.06	2.23*	0.04	1.82	0.08	2.13*	0.04
**Ch 45**	29	2.14*	0.04	2.32*	0.03	2.05*	0.05	2.83*	0.01
**Ch 46**	27	-0.53	0.60	-0.65	0.52	0.24	0.81	0.04	0.97
**Ch 47**	21	0.21	0.84	0.14	0.89	0.58	0.57	0.72	0.48
**Ch 48**	-	-	-	-	-	-	-	-	-

Supplementary Table 4. Group level t-values maps for the contrast Mixed>Go-only for HbR for the CB task. Results are reported for pipeline 1 with a band-pass filter with cut-off frequencies [0.01 0.5 Hz] and [0.01 0.1] Hz, pipeline 2 regressing out the short separation channel with the highest correlation (SSR correlation) and pipeline 2 regressing out the average of all short separation channels (SSR average). Single asterisks indicate significant channels at $p<0.05$ uncorrected; double asterisks indicate significant channels surviving false discovery rate multiple comparison correction at $p<0.05$. A positive t-value corresponds to a larger HbR decrease in the Mixed blocks compared to the Go-only blocks.

HbR		Pipeline 1 with [0.01 0.5] Hz filter		Pipeline 1 with [0.01 0.1] Hz filter		Pipeline 2 SSR correlation		Pipeline 2 SSR average	
	df	t-value	p-value	t-value	p-value	t-value	p-value	t-value	p-value
**Ch 1**	23	0.13	0.90	-0.02	0.98	-0.34	0.74	0.03	0.98
**Ch 2**	27	-0.74	0.47	-0.75	0.46	-1.26	0.22	-1.02	0.32
**Ch 3**	-	-	-	-	-	-	-	-	-
**Ch 4**	19	1.03	0.32	0.90	0.38	0.95	0.35	0.97	0.34
**Ch 5**	12	-0.04	0.97	-0.03	0.98	0.28	0.78	-0.46	0.65
**Ch 6**	26	-1.25	0.22	-1.22	0.23	-1.37	0.18	-0.52	0.61
**Ch 7**	24	-0.92	0.37	-1.06	0.30	-1.31	0.20	-0.87	0.39
**Ch 8**	29	-0.64	0.53	-0.58	0.57	-0.88	0.39	-0.09	0.93
**Ch 9**	25	0.07	0.94	-0.21	0.83	-0.18	0.86	-0.14	0.89
**Ch 10**	14	-0.54	0.60	-0.49	0.63	-0.91	0.38	-0.49	0.63
**Ch 11**	22	-3.28*	0.003	-2.85*	0.01	-2.92*	0.01	-3.04*	0.01
**Ch 12**	21	-0.82	0.42	-0.83	0.42	-1.21	0.24	-1.09	0.29
**Ch 13**	15	0.75	0.46	0.63	0.54	-0.27	0.79	0.38	0.71
**Ch 14**	-	-	-	-	-	-	-	-	-
**Ch 15**	26	-0.10	0.92	-0.10	0.92	0.01	0.99	-0.25	0.81
**Ch 16**	25	0.26	0.80	0.19	0.85	0.52	0.61	0.97	0.34
**Ch 17**	21	0.56	0.58	0.44	0.66	0.62	0.54	0.59	0.56
**Ch 18**	14	-0.61	0.55	-0.64	0.53	0.03	0.97	-0.52	0.61
**Ch 19**	19	0.02	0.99	-0.10	0.92	0.01	0.99	-0.48	0.64
**Ch 20**	15	-2.30*	0.04	-2.28*	0.04	-2.45*	0.03	-2.82*	0.01
**Ch 21**	22	0.12	0.90	0.12	0.91	0.00	1.00	0.32	0.75
**Ch 22**	24	1.23	0.23	1.17	0.25	0.79	0.44	1.63	0.12
**Ch 23**	24	1.62	0.12	1.63	0.12	0.82	0.42	0.81	0.43
**Ch 24**	20	2.39*	0.03	2.47*	0.02	2.60*	0.02	2.98*	0.01
**Ch 25**	16	0.42	0.68	0.39	0.70	-0.55	0.59	0.13	0.90
**Ch 26**	26	1.75	0.09	1.67	0.11	1.10	0.28	1.64	0.11
**Ch 27**	15	-1.08	0.30	-1.33	0.20	-2.47*	0.03	-1.27	0.22
**Ch 28**	14	-0.67	0.51	-0.69	0.50	-0.92	0.37	-1.04	0.32
**Ch 29**	19	0.76	0.46	0.75	0.46	0.80	0.43	0.97	0.35
**Ch 30**	15	1.35	0.20	1.23	0.24	1.19	0.25	1.18	0.26

**Ch 31**	21	1.31	0.20	1.19	0.25	1.34	0.19	1.97	0.06
**Ch 32**	21	1.73	0.10	1.70	0.10	1.72	0.10	1.91	0.07
**Ch 33**	13	-0.12	0.91	-0.20	0.84	0.08	0.94	-0.41	0.69
**Ch 34**	16	-0.09	0.93	-0.22	0.83	-0.02	0.98	-0.19	0.85
**Ch 35**	20	0.11	0.91	0.09	0.93	0.47	0.64	-0.05	0.96
**Ch 36**	10	-1.58	0.14	-1.74	0.11	-1.34	0.21	-1.65	0.13
**Ch 37**	-	-	-	-	-	-	-	-	-
**Ch 38**	25	2.16*	0.04	2.03	0.05	1.59	0.12	1.80	0.08
**Ch 39**	25	0.76	0.45	0.52	0.61	0.39	0.70	0.68	0.51
**Ch 40**	26	0.20	0.84	0.07	0.94	-0.27	0.79	0.30	0.76
**Ch 41**	14	0.28	0.78	0.11	0.92	-0.57	0.58	-0.44	0.67
**Ch 42**	25	1.35	0.19	1.21	0.24	1.08	0.29	1.85	0.08
**Ch 43**	17	-0.73	0.48	-0.80	0.44	-1.43	0.17	-0.60	0.56
**Ch 44**	25	-0.48	0.64	-0.59	0.56	-0.32	0.75	-0.62	0.54
**Ch 45**	29	1.43	0.16	1.33	0.20	0.92	0.36	1.34	0.19
**Ch 46**	27	0.87	0.39	0.94	0.36	0.24	0.81	0.93	0.36
**Ch 47**	21	-1.09	0.29	-1.33	0.20	-1.53	0.14	-1.14	0.27
**Ch 48**	-	-	-	-	-	-	-	-	-

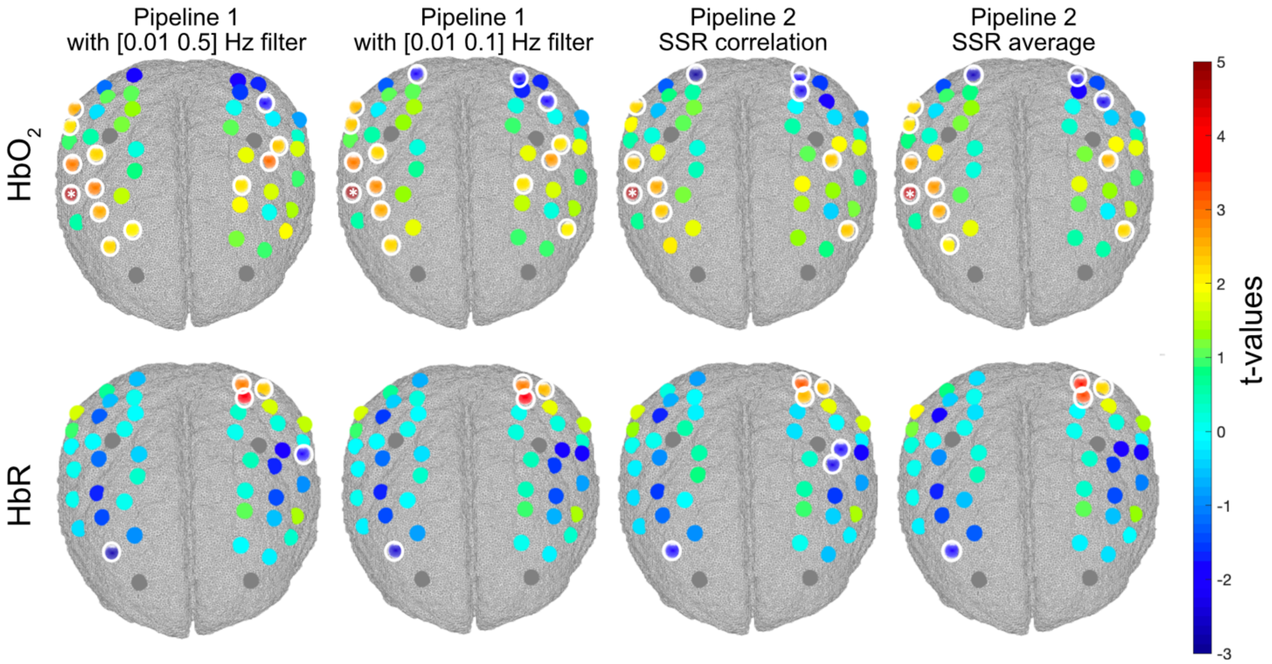

Supplementary Figure 3. Group level t-values maps for the contrast Mixed>Go-only for the VR task across different pipelines. Statistically significant channels at $p<0.05$ are circled in white. Channels surviving FDR correction are marked with asterisks. A positive t-value corresponds to a HbO_2 increase and a HbR decrease; a negative t-value corresponds to a HbO_2 decrease and a HbR increase. Results are shown for pipeline 1 with a band-pass filter with cut-off frequencies [0.01 0.5 Hz] and [0.01 0.1] Hz, pipeline 2 regressing out the short separation channel with the highest correlation (SSR correlation) and pipeline 2 regressing out the average of all short separation channels (SSR average).

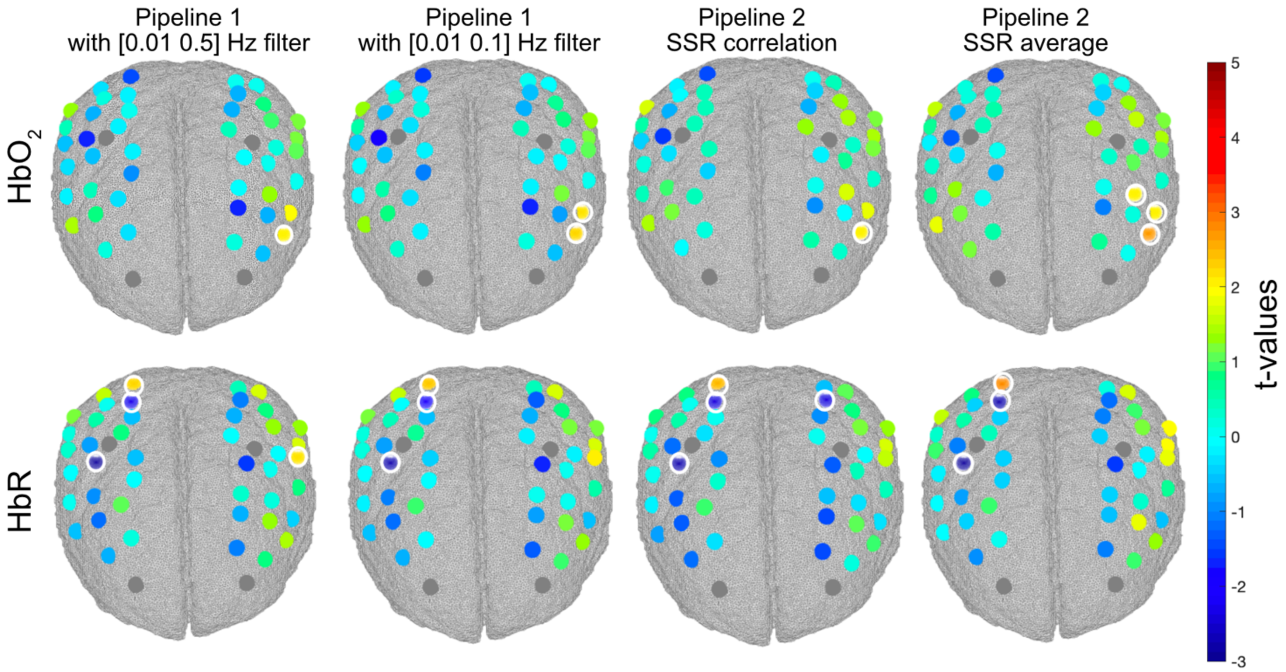

Supplementary Figure 4. Group level t-values maps for the contrast Mixed>Go-only for the CB task across different pipelines. Statistically significant channels at $p<0.05$ are circled in white. Channels surviving FDR correction are marked with asterisks. A positive t-value corresponds to a HbO_2 increase and a HbR decrease; a negative t-value corresponds to a HbO_2 decrease and a HbR increase. Results are shown for pipeline 1 with a band-pass filter with cut-off frequencies [0.01 0.5 Hz] and [0.01 0.1] Hz, pipeline 2 regressing out the short separation channel with the highest correlation (SSR correlation) and pipeline 2 regressing out the average of all short separation channels (SSR average).

3. Group-level statistical analysis results for the contrast of interest Mixed>Go-only across different pipelines

Supplementary Table 5. Group level t-values maps for the contrasts Go-only>0, Mixed>0, Mixed>Go-only for the short separation channels. Single asterisks indicate significant channels at p<0.05 uncorrected.

			Go-only > 0				Mixed > 0				Mixed > Go-only			
			HbO_2		HbR		HbO_2		HbR		HbO_2		HbR	
		df	t-value	p-value	t-value	p-value	t-value	p-value	t-value	p-value	t-value	p-value	t-value	p-value
VR	Ch 3	24	-1.41	0.17	-0.78	0.44	-0.81	0.42	0.24	0.81	0.46	0.65	0.49	0.63
	Ch 14	12	-1.46	0.17	2.14	0.05	0.96	0.36	-0.27	0.79	1.91	0.08	-2.18	0.05
	Ch 37	14	0.58	0.57	0.37	0.72	-0.60	0.56	1.27	0.22	-0.81	0.43	0.80	0.43
	Ch 48	21	0.16	0.87	-0.86	0.40	-2.54*	0.02	0.99	0.33	-1.72	0.10	1.13	0.27
CB	Ch 3	23	1.05	0.30	0.83	0.42	0.52	0.61	0.87	0.39	-0.75	0.46	-0.63	0.53
	Ch 14	13	2.27*	0.04	0.23	0.82	-0.60	0.56	0.63	0.54	-2.09	0.06	0.30	0.77
	Ch 37	14	0.87	0.40	0.59	0.57	-0.75	0.46	-1.00	0.33	-1.05	0.31	-0.76	0.46
	Ch 48	19	2.36*	0.03	-1.58	0.13	-1.50	0.15	1.87	0.08	-2.24*	0.04	2.01	0.06
